# Automated classification of stress and relaxation responses in major depressive disorder, panic disorder, and healthy participants via heart rate variability

**DOI:** 10.3389/fpsyt.2024.1500310

**Published:** 2025-01-09

**Authors:** Sangwon Byun, Ah Young Kim, Min-Sup Shin, Hong Jin Jeon, Chul-Hyun Cho

**Affiliations:** ^1^ Department of Electronics Engineering, Incheon National University, Incheon, Republic of Korea; ^2^ Medical Information Research Section, Electronics and Telecommunications Research Institute, Dajeon, Republic of Korea; ^3^ Department of Psychology, Korea University, Seoul, Republic of Korea; ^4^ Department of Psychiatry, Depression Center, Samsung Medical Center, Sungkyunkwan University School of Medicine, Seoul, Republic of Korea; ^5^ Meditrix Co., Ltd., Seoul, Republic of Korea; ^6^ Department of Psychiatry, Korea University College of Medicine, Seoul, Republic of Korea; ^7^ Department of Biomedical Informatics, Korea University College of Medicine, Seoul, Republic of Korea

**Keywords:** heart rate variability, major depressive disorder, panic disorder, stress, relaxation, machine learning, autonomic nervous system, physiological signals

## Abstract

**Background:**

Stress is a significant risk factor for psychiatric disorders such as major depressive disorder (MDD) and panic disorder (PD). This highlights the need for advanced stress-monitoring technologies to improve treatment. Stress affects the autonomic nervous system, which can be evaluated via heart rate variability (HRV). While machine learning has enabled automated stress detection via HRV in healthy individuals, its application in psychiatric patients remains underexplored. This study evaluated the feasibility of using machine-learning algorithms to detect stress automatically in MDD and PD patients, as well as healthy controls (HCs), based on HRV features.

**Methods:**

The study included 147 participants (MDD: 41, PD: 47, HC: 59) who visited the laboratory up to five times over 12 weeks. HRV data were collected during stress and relaxation tasks, with 20 HRV features extracted. Random forest and multilayer perceptron classifiers were applied to distinguish between the stress and relaxation tasks. Feature importance was analyzed using SHapley Additive exPlanations, and differences in HRV between the tasks (ΔHRV) were compared across groups. The impact of personalized longitudinal scaling on classification accuracy was also assessed.

**Results:**

Random forest classification accuracies were 0.67 for MDD, 0.69 for PD, and 0.73 for HCs, indicating higher accuracy in the HC group. Longitudinal scaling improved accuracies to 0.94 for MDD, 0.90 for PD, and 0.96 for HCs, suggesting its potential in monitoring patients’ conditions using HRV. The HC group demonstrated greater ΔHRV fluctuation in a larger number of and more significant features than the patient groups, potentially contributing to higher accuracy. Multilayer perceptron models provided consistent results with random forest, confirming the robustness of the findings.

**Conclusion:**

This study demonstrated that differentiating between stress and relaxation was more challenging in the PD and MDD groups than in the HC group, underscoring the potential of HRV metrics as stress biomarkers. Psychiatric patients exhibited altered autonomic responses, which may influence their stress reactivity. This indicates the need for a tailored approach to stress monitoring in these patient groups. Additionally, we emphasized the significance of longitudinal scaling in enhancing classification accuracy, which can be utilized to develop personalized monitoring technologies for psychiatric patients.

## 1 Introduction

Psychiatric disorders are increasingly common worldwide and present significant global health challenges (1–3). The most prevalent psychiatric disorders include major depressive disorder (MDD) and anxiety disorders, which affect over 250 million and 300 million people worldwide, respectively (4, 5). MDD is characterized by a persistently depressed mood or loss of interest in activities, along with symptoms such as weight changes, sleep disturbances, fatigue, and feelings of worthlessness, making it a leading cause of global disability (6, 7). Panic disorder (PD) is a common anxiety disorder that involves recurrent, unexpected panic attacks with intense fear and symptoms, such as heart palpitations and sweating, and persistent worry about future attacks or behavioral changes to avoid them, all of which disrupt functions of daily life (7, 8). Left untreated, these debilitating mental illnesses severely impair cognitive function, reduce quality of life, and, in some cases, lead to suicide, which substantially contributes to their global burden (1–3).

Previous research has indicated that stress is associated with an increased risk of developing and exacerbating MDD and PD (9, 10). Specifically, both chronic and acute stress have significant associations with the onset of clinical episodes of depression and PD (10–14). Prolonged exposure to stressors has been linked to a more refractory course of MDD and PD (15). Additionally, acute stressful events can trigger the recurrence of depression (16). Therefore, developing technologies to evaluate the severity and persistence of stress exposure through individual patient monitoring is necessary to improve the treatment of these disorders.

Stress affects the autonomic nervous system (ANS), responsible for regulating physiological responses to external stimuli (17–19). The ANS typically presents increased sympathetic activity and withdrawn parasympathetic activity in response to stress (17–19). Increasing research has explored methods to assess stress by quantifying these autonomic responses (20). Heart rate variability (HRV), which reflects the variations in the time intervals between heartbeats, is an extensively studied measure. It is indicative of cardiac autonomic regulation mediated by both the sympathetic and parasympathetic nervous systems (17–19). HRV is recognized as a quantitative biomarker for evaluating ANS function and its responses to physiological and environmental stimuli (21). Additionally, mobile technological advancement has led to the use of wearable devices as non-invasive approaches to monitor stress based on HRV (22). Previous studies have established that the autonomic response to stress, manifested as reduced HRV, leads to detectable changes in physiological signals, which is captured by wearable devices (22).

Accordingly, recent studies have utilized machine-learning techniques to automatically detect stress based on HRV (21, 23). Various machine-learning methods, from classical to deep learning algorithms, have implemented automated stress detection based on HRV and demonstrated successful performance in classifying stress (21, 23). However, these studies have focused on detecting stress in healthy individuals rather than patients with psychiatric disorders. Particularly, stress analysis based on HRV in patients with psychiatric conditions has focused on how patients responded to stress differently compared with healthy controls (HCs) and relied on statistical methods.

Psychiatric disorders have been associated with ANS dysfunction, which can lead to autonomic imbalance toward sympathetic activation, as reflected in HRV (24–26). MDD patients in particular often show altered autonomic regulation that affects cardiovascular control, with decreased cardiac vagal modulation (27). Consequently, patients with MDD and PD typically exhibit lower HRV compared with HCs, which indicates reduced autonomic flexibility (24–26). This altered autonomic response in patients causes differences in stress reactions between patients and healthy individuals. Patients with MDD exhibited lower reactivity to stress than HCs, evidenced by lower fluctuations in their HRV (28). Research in patients with PD revealed mixed stress responses and reported higher (29), reduced (30), and similar reactivity (31) compared with HCs. Although previous studies compared stress responses via HRV between patients and healthy individuals, research on the application of machine learning to identify stressful states in psychiatric patients based on HRV data is lacking.

Our study aimed to explore the feasibility of automated stress detection based on HRV features via machine-learning algorithms in patients with MDD and PD, as well as HCs. HRV features were obtained from three distinct participant groups: MDD, PD, and HC, while they performed various experimental tasks, which included those designed to induce mental stress and relaxation. We focused on distinguishing between the states of stress and relaxation via HRV features and compared the classification results across different participant groups. We hypothesize that machine-learning algorithms can effectively classify stress and relaxation states based on HRV features, with the accuracy potentially differing among three groups, namely, MDD, PD, and HC, due to varying ANS responses. Notably, mental disorders, such as MDD and PD, demonstrated substantial individual variability among patients, a characteristic that reflected the heterogeneous nature of these conditions (32, 33). Therefore, we investigated the impact of individually scaling patient data on the classification outcomes as a pilot study. We believe that this approach could facilitate the development of further precise and automated methods for monitoring stress in patients with psychiatric problems and ultimately lead to improved management and treatment strategies.

## 2 Methods

### 2.1 Participants

Participants included 147 individuals: 41 with MDD, 47 with PD, and 59 HCs. All patients were recruited at the Samsung Medical Center in Seoul, Korea, between December 2015 and January 2017 (34). MDD and PD diagnoses were conducted by a senior psychiatrist in accordance with Diagnostic and Statistical Manual of Mental Disorder, Fifth Edition (DSM-V) criteria (7). Exclusion criteria included pregnancy, history of substance or alcohol abuse, head injury, high suicide risk, personality disorders, severe physical ailments, and long-acting medication use (e.g., fluoxetine and depot neuroleptics). All patients received standard psychiatric pharmacotherapy for MDD or PD throughout the duration of the 12-week experiment, which included standard antidepressant treatments, such as selective serotonin reuptake inhibitors (SSRIs), serotonin norepinephrine reuptake inhibitors (SNRIs), norepinephrine dopamine reuptake inhibitors, and tricyclic antidepressants (TCAs) (34). HCs who lacked a psychiatric history or family background of mood disorders were recruited through general study advertisements. The study protocol was approved by the Ethics Committee of Samsung Medical Center in Seoul, Korea (No. 2015-07-151), and complied with the applicable guidelines. All participants provided written informed consent after they received a thorough explanation of the research procedures. Additionally, each participant received $50 as compensation.

### 2.2 Study design

**Figure 1:**
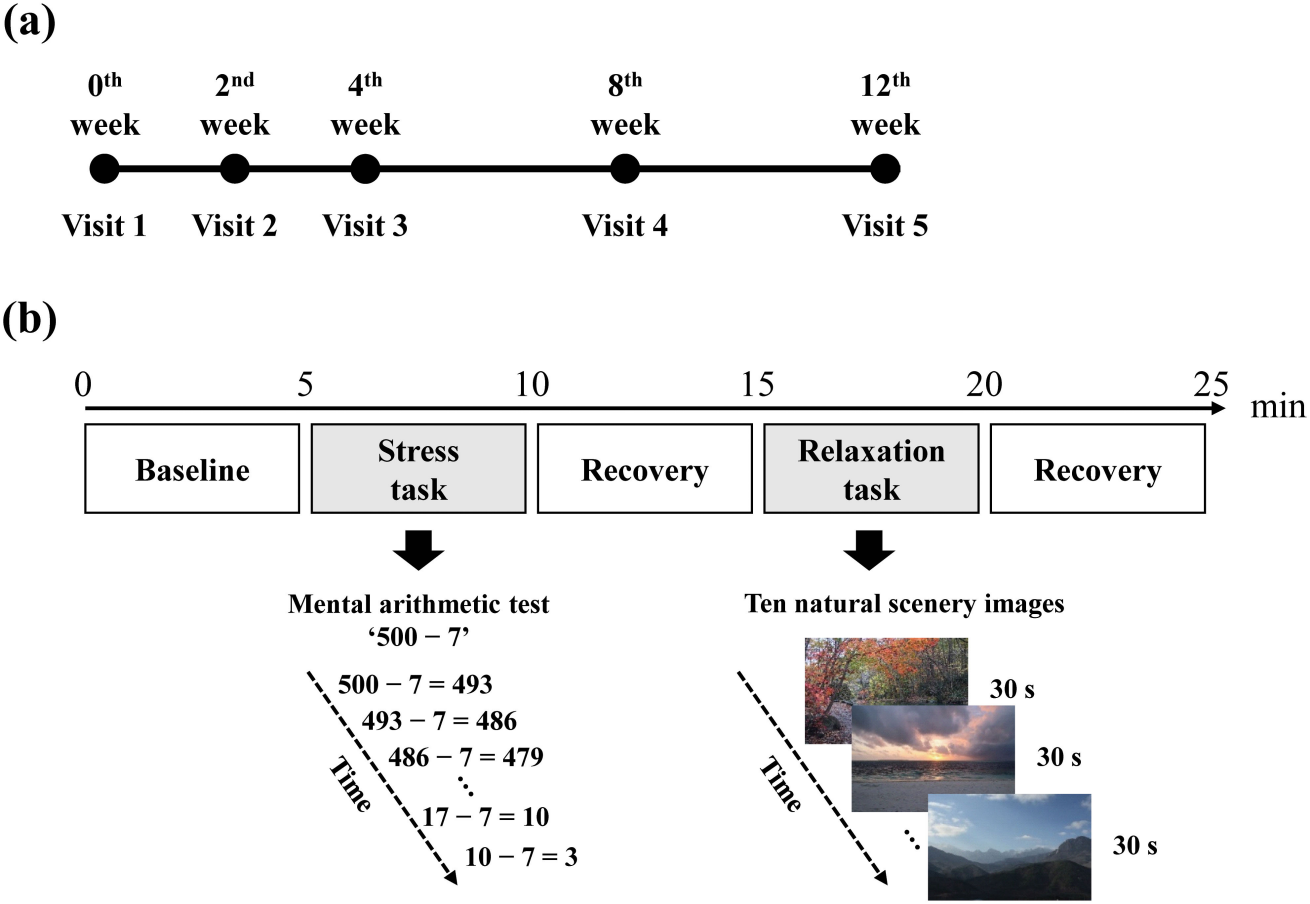
Experimental protocol. **(A)** The study lasted for 12 weeks, with each participant scheduled for a total of five visits. **(B)** During each visit, ECG signals were recorded in five consecutive phases, with each phase lasting for 5 minutes.

The study spanned 12 weeks for each participant (**Figure 1A**), with five scheduled visits to our clinical laboratory: baseline and subsequent visits at 2, 4, 8, and 12 weeks. Each participant provided demographic information (e.g., age and sex) and underwent clinical evaluations. Clinical evaluations incorporated the Hamilton rating scale for depression (HAMD), Hamilton rating scale for anxiety (HAMA), and panic disorder severity scale (PDSS), which were administered during the initial and 12-week visits (35–37). Participants’ body mass index (BMI) was also assessed, considering its recognized influence on ANS responses (38). This study is part of a larger investigation examining changes in clinical symptoms and inflammatory biomarkers over 12 weeks to capture treatment effects (39).

### 2.3 Experimental protocol

The experimental procedure was developed to examine autonomic responses to stress and relaxation tasks. The protocol comprised five phases, each lasting five minutes, totaling to a duration of 25 minutes. Furthermore, physiological signals, such as electrocardiograms (ECG), were continuously measured while the participants performed specific tasks in each phase (**Figure 1B**). The first phase, serving as the baseline phase, involved a rest period during which the participants were instructed to sit comfortably and minimize movement. In the second phase, the participants undertook a stress task involving a mental arithmetic test (MAT), during which they were required to subtract serial 7s starting from 500 and verbally report their answers to the researchers. The participants were prompted to recalculate in case an error occurred. If the participants reached the final answer, 10 minus 7 equals 3, before the 5-minute phase ended, they restarted the task from 500 and continued subtracting. The third phase, also a rest phase, involved participants discontinuing arithmetic calculations and resting, which allowed autonomic recovery from the stress task. In the fourth phase, the participants performed a relaxation task by observing 10 consecutive images of natural scenery on a computer screen, each displayed for 30 seconds. The final phase, another rest phase, involved resting without any image presentation to allow recovery from the relaxation task. Two trained investigator specialists conducted the experiments. Only one participant was examined at a time by a specialist in our clinical laboratory. In our study, the sequence of stress and relaxation tasks was not randomized. As randomizing the order could reduce potential biases in the results, we plan to implement this approach in future research.

The MAT task used in this study was specifically designed to induce cognitive and psychological stress by progressively increasing participants’ mental load through continuous subtraction tasks (40–43). Research has demonstrated that MAT effectively induces physiological changes, including alterations in heart rate, skin conductance response, and neural activity (40–43). In our prior studies, we similarly observed a significant decrease in HRV when using the same stimulus, as compared to baseline measurements (44). Additionally, research has shown that exposure to nature scenes, which served as the relaxation task in this study, positively supports autonomic recovery from stress, as assessed by HRV and skin conductance measurements (45, 46).

### 2.4 Physiological measurement

We recorded physiological signals during working hours, considering the potential influence of the participant’s physiological state, which included factors such as time of day, mood, and rest (47–49). The experiment was conducted in a controlled environment, specifically a sound-attenuated room maintained at a temperature of 23°C and humidity levels between 45%–55%. Participants were instructed to sit comfortably in an armchair with a headrest prior to the experiment and avoid unnecessary movement or speech while the devices to record their physiological signals were being set up and calibrated. ECG signals were collected via the ProComp Infiniti system (SA7500, Thought Technology, Canada) at a sampling rate of 256 Hz, chosen to ensure an accurate analysis of the QRS complex and R-peak (50). ECGs were captured with an ECG-Flex/Pro sensor (T9306M, Thought Technology), with three electrodes placed on both forearms: the negative lead on the right forearm and positive and ground leads on the left forearm. The collected ECG signals were filtered using a 60 Hz notch filter provided in the BioGraph Infiniti software (Thought Technology).

R-peak to R-peak interval (RRI) data from the ECG signals were analyzed via Kubios HRV Premium software (Kubios, www.kubios.com), which utilized an in-house-developed QRS detection algorithm based on the Pan-Tompkins method (51, 52). The RRI data underwent visual inspection, and any artifacts were rectified via a piecewise cubic spline interpolation method. The entire analysis was performed by the same operator to ensure consistency. Subsequently, the HRV features were calculated separately from the RRI data of the individual phases.

### 2.5 HRV feature extraction

A standard HRV analysis was conducted according to international guidelines (50, 53). We derived 20 HRV features from the RRI data of each phase and covered time, frequency, and nonlinear domain analyses (**Supplementary Table 1**). Time and frequency domains are traditional approaches widely used in numerous studies, demonstrating well-established connections with the ANS (50, 53). The nonlinear domain has gained attention more recently and is increasingly being recognized for its potential as a biomarker. Nonlinear features are now being utilized not only to assess autonomic responses to external stimuli, such as stress, but also in the context of mental health conditions (54, 55). In this study, we included the most representative features of these three domains.

Time-domain HRV features were directly calculated from the RRI time series. We extracted six features via this analysis: the mean of the RRIs, standard deviation of the RRIs (SDNN), root mean square of successive RRI differences (RMSSD), percentage of successive RRIs differing by more than 50 ms (pNN50), integral of the histogram of the RRI divided by its height (TRI), and baseline width of the RRI histogram (TINN). Seven features were calculated via the frequency domain analysis. The RRI data were converted to equidistantly sampled data via cubic spline interpolation (4 Hz). Power spectral density was estimated via Welch’s periodogram-based fast Fourier transform. Absolute powers were computed in very low-frequency (VLF, 0–0.04 Hz), low-frequency (LF, 0.04–0.15 Hz), and high-frequency (HF, 0.15–0.4 Hz) bands. Additionally, the relative powers of the LF and HF bands in normalized units and the LF/HF power ratio were calculated. Absolute powers were expressed in natural logarithms to reduce skewness in the distribution.

We extracted five nonlinear measures to assess the nonlinear dynamics in heart rate signals. Approximate entropy (ApEn) measured the irregularity in short and noisy time-series data and did not assume underlying system dynamics (56). The embedding dimension and tolerance value for ApEn were set to 2 and 0.2, respectively. Sample entropy (SampEn) was developed to reduce ApEn bias from self-comparison and was more reliable for shorter time series, with parameters set identical to those for ApEn (57). Detrended fluctuation analysis (DFA) was used to assess fractal scaling properties of short-term RRI signals by integrating and detrending the time-series data and subsequently measured the root-mean-square fluctuation at different time scales (58). The fluctuation was defined by α1 and α2, which represented short-range and long-range correlations, respectively. In this study, α1 and α2 were evaluated for data lengths of 4–16 and 17–64, respectively. The correlation dimension (CorDim) estimated the number of independent variables required to model the signal, and higher values indicated greater complexity (59, 60). We derived two features from the Poincaré plot analysis, which graphically represented the correlation between successive RRIs. SD1 and SD2 represented the standard deviations perpendicular to and along the line of identity, respectively.

### 2.6 Statistical analyses

Statistical analyses were conducted using SPSS version 25 (SPSS Inc., Chicago, IL, USA). Demographic and clinical characteristics from the MDD, PD, and HC groups were compared via the one-way analysis of variance (ANOVA), except for sex, which was compared via a chi-square test. HRV features among the MDD, PD, and HC groups measured during the stress and relaxation tasks, were compared via one-way ANOVA on mean values from all five visits. We conducted within-subject comparisons of HRV features between the stress and relaxation tasks during a single visit via paired samples t-tests. Differences in HRV features between stress and relaxation tasks, defined as ΔHRV, were calculated within the same participants during a single visit. We compared ΔHRV among the MDD, PD, and HC groups via one-way ANOVA. For all one-way ANOVAs reported in this study, we employed either Fisher’s ANOVA with Bonferroni *post-hoc* analysis or Welch’s ANOVA with Games-Howell *post-hoc* analysis based on the homogeneity of variance. A *P* value < 0.05 was considered statistically significant. We chose a one-way ANOVA to focus specifically on the differences in HRV across the three groups, rather than on variations introduced by factors such as visit. This approach allowed us to emphasize the primary objective of understanding HRV differences among diagnostic groups. Future studies may incorporate additional factors in a more comprehensive model.

### 2.7 Classification of the stress and relaxation tasks

To classify the stress and relaxation tasks based on HRV features, we implemented two machine-learning algorithms: random forest and multilayer perceptron (MLP). Although 735 samples were expected if 147 participants (41 MDD patients, 47 PD patients, and 59 HCs) visited five times each, some participants missed visits. Consequently, 650 samples were obtained each for stress and relaxation (181 MDD, 191 PD, and 278 HC). Hence, 1300 samples were used for classification. All classifications were performed with Python version 3.11.4 (Python Software Foundation).

We utilized 20 HRV features as input data. Training data were normalized by subtracting the mean and dividing it by the standard deviation. Subsequently, the same statistical values were used to normalize the test dataset. However, this normalization was not applied when we conducted personalized longitudinal scaling. The stress and relaxation tasks were defined as the positive and negative class for classification, respectively.

We used a stratified 10-fold cross-validation (CV) repeated 20 times to evaluate performance measures of classification (**Supplementary Figure 1**). The task was used as a stratification option. A subject-wise split was used to ensure that all data from a given participant was contained entirely within either the training or the test set, not both, to avoid data leakage. Nine folds were used for training, and the remaining fold was used for evaluation. We created 10 models and evaluated for each fold. We averaged the results from 10 folds to estimate the model’s performance. This entire process was repeated 20 times. Therefore, performance metrics were presented as the mean and standard deviation calculated from 20 repeats. Performance indices included accuracy, F1, recall, precision, and area under the curve (AUC).

Sample sizes for the MDD, PD, and HC groups were 362, 382, and 556, respectively. Despite the variations in sample sizes, we initially conducted the classification without matching the sample sizes. However, we later repeated the classification via the same method after matching the sample sizes. We employed random undersampling to match the sample sizes and aligned them with the smallest sample size, which belonged to the MDD group.

Moreover, we built models trained and tested exclusively on data from one group. The entire dataset was divided into three separate datasets for the MDD, PD, and HC groups. Subsequently, three separate models were trained and tested, each using the data from one specific group exclusively, which ensured that data from different groups did not interact during the training.

### 2.8 Random forest and MLP classifiers

We selected the random forest algorithm owing to its capacity to effectively manage non-linear relationships and high-dimensional feature spaces and its ability to provide feature importance evaluations (61, 62). We utilized this algorithm to compute SHapley Additive exPlanations (SHAP) values and subsequently conducted an analysis of the model’s classification results based on these values. We performed hyperparameter optimization using grid search within the training set with a 5-fold CV, ensuring optimal model performance while preventing data leakage into the test set. The number of trees, a key hyperparameter in the random forest algorithm, was optimized using the following values: 50, 100, and 200.

We repeated the classification via MLP with the same approach as that for random forest. This was to ensure that our results were not algorithm-dependent and demonstrate consistency across different algorithms. MLP was chosen as it was based on neural networks, which offered a completely different classification method compared with the ensemble-based random forest. This approach helped us verify the robustness and reliability of our findings across diverse machine-learning techniques. The following hyperparameters were optimized using the same approach as applied to the random forest: hidden layer sizes of (4, 8, 16) and (4, 8, 16, 32), as well as initial learning rates of 0.0001, 0.001, and 0.01. A total of six combinations were explored using the grid search method. Accordingly, we evaluated MLP architectures with three- and four-hidden-layer configurations (**Supplementary Figure 2**). All hidden layers were dense layers and used ReLU as the activation function. The output layer used sigmoid as the activation function to perform binary classification. Dropout was not used. Adam optimizer was the solver. We applied an L2 penalty with a coefficient of 0.0001 for regularization. Furthermore, we had set the MLP model with a maximum of 1000 iterations and enabled early stopping. The training was stopped if the validation score did not improve by at least 10^-4^ for 10 consecutive iterations.

In this study, we did not conduct feature selection separately. The random forest algorithm inherently performs a form of feature selection, since it constructs multiple decision trees, each trained on a random subset of features (63). In contrast, it should be considered that the use of an MLP could benefit from feature selection to improve model performance (64). However, our study utilized over 1000 samples to train a model with 20 features, leading to a sample-to-feature ratio that we considered sufficient. Consequently, we concluded that feature selection was not strictly necessary for this dataset.

### 2.9 Model interpretation via SHAP

SHAP values were calculated via random forest on test datasets to interpret classification outcomes (65, 66). SHAP, based on Shapley values, utilized cooperative game theory developed by Lloyd Shapley (67). The SHAP value quantified the impact of each input feature on predicting the output for each individual (68). Our analysis involved a 10-fold CV repeated 20 times, and the reported SHAP values represented the averages across the 20 iterations of the 10-fold CV.

### 2.10 Personalized longitudinal scaling

**Figure 2:**
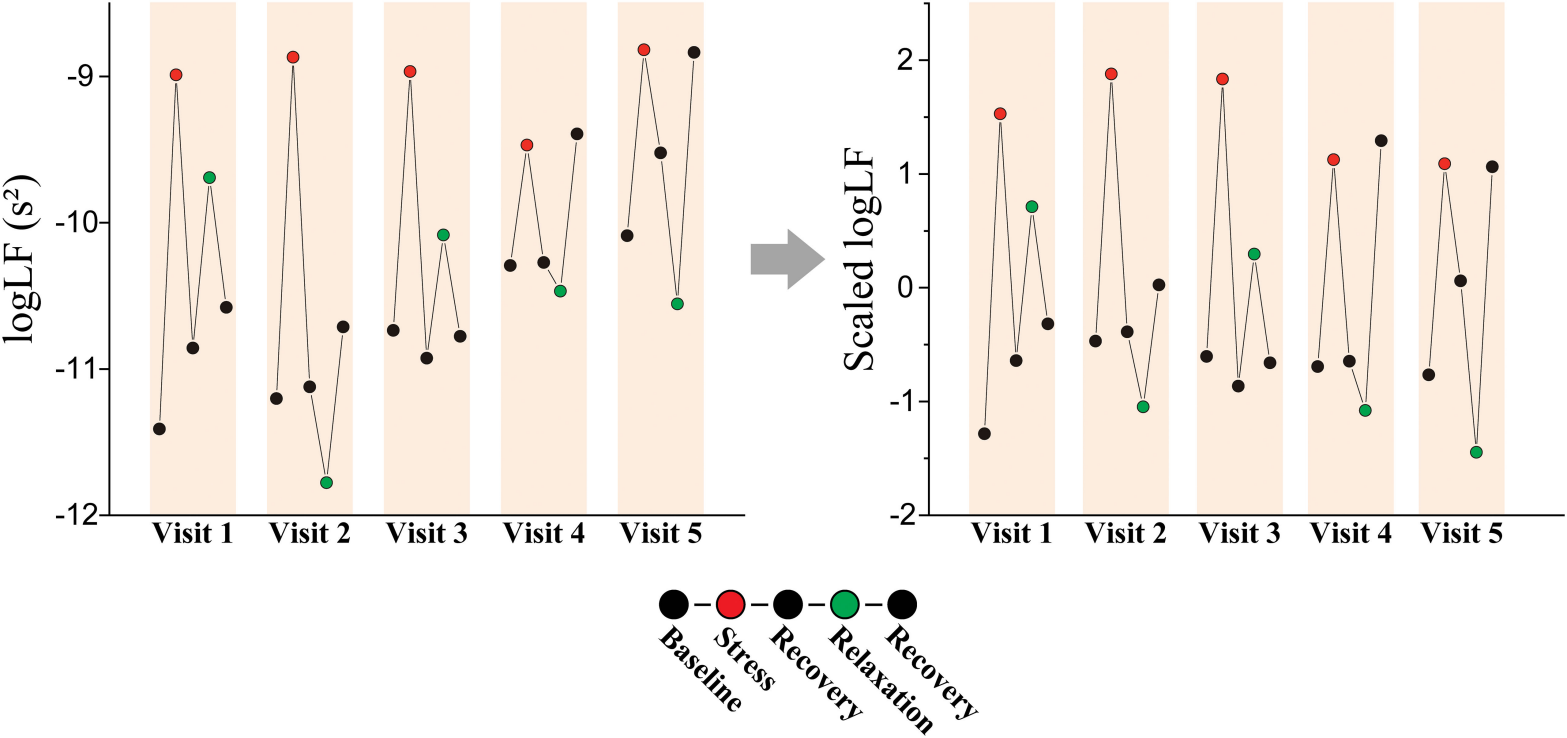
Personalized longitudinal scaling. An example of scaling on logLF measured from a patient with MDD (female, 61-year-old).

Our participants attended up to five visits over a 12-week span and completed five tasks per visit. This approach allowed for data collection at multiple time points for each individual. To utilize this advantage, data for each participant were normalized over the time axis (**Figure 2**). We utilized all the data from these visits and tasks for personalized longitudinal scaling, considering extensive data while subjecting participants to various experimental conditions. Means and standard deviations were calculated via the data measured from a single participant. Subsequently, the data from this participant were normalized by subtracting the mean and dividing by the standard deviation. We repeated this process individually for each participant. We performed classification analyses via the scaled HRV data to evaluate whether personalized longitudinal scaling enhanced the classification of stress versus relaxation responses and applied the same methodologies.

Furthermore, we applied t-stochastic neighbor embedding (t-SNE) to the HRV data both before and after personalized longitudinal scaling to evaluate its impact The t-SNE was a machine-learning technique designed to visualize high-dimensional data by projecting it into a low-dimensional space (69). It aimed to maintain the relative similarity between data points from the original high-dimensional space in the resulting low-dimensional representation. Projection was determined by minimizing the Kullback-Leibler-divergence between the similarity of data distributions in the high- and low-dimensional space (70). We conducted t-SNE using 5000 iterations with a perplexity of 50.

## 3 Results

### 3.1 Demographic and clinical characteristics

Our participants included 41 (30 females) and 47 (30 females) patients with MDD and PD, respectively, and 59 HCs (36 females). **Table 1** summarizes the participants’ demographic and clinical characteristics. No significant differences were observed in age, sex, or BMI among the groups, which indicated balanced participants and reduced the potential confounding effects of these variables on HRV outcomes. The MDD and PD groups showed significantly higher HAMD and HAMA scores than the control group, which reflected the expected clinical severity of depressive and anxiety symptoms (**Supplementary Table 2**). PDSS score was highest in the PD group, followed by the MDD group, and lowest in the HC group, which aligned with the diagnostic criteria and expected symptomatology of these groups.

**Table 1:** Demographic and clinical characteristics of the MDD, PD, and HC groups.

	MDD (N = 41)	PD (*N* = 47)	HC (*N* = 59)	*F or χ* ^2^ (*P* value)	*Post-hoc*
Demographic data					
Age (years)	42.02 ± 16.65	41.64 ± 14.39	38.49 ± 14.22	0.88 (.42)^a^	ns
Sex (M/F)	11/30	17/30	23/36	1.64 (.44)	ns
BMI (kg/m^2^)	22.93 ± 3.41	23.29 ± 3.26	22.76 ± 3.17	0.35 (.71)^a^	ns
Clinical data					
HAMD	17.49 ± 7.07	13.87 ± 7.71	1.88 ± 1.75	143.26 (<.001)^b^	MDD, PD > HC^b^
HAMA	16.56 ± 8.46	15.11 ± 8.44	2.12 ± 2.22	103.53 (<.001)^b^	MDD, PD > HC^b^
PDSS	3.61 ± 5.74	12.53 ± 6.05	0.02 ± 0.13	107.16 (<.001)^b^	PD > MDD > HC^b^

Data are presented as means and standard deviations for continuous variables and as counts for categorical variables. See **Supplementary Table 2** for *post-hoc P* values.

ns, No significant main effect; MDD, major depressive disorder; PD, panic disorder; HC, healthy control; BMI, body mass index; HAMD, Hamilton rating scale for depression; HAMA, Hamilton rating scale for anxiety; PDSS, panic disorder severity scale.

a Fisher’s one-way ANOVA.

b Welch’s one-way ANOVA and Games-Howell *post-hoc* analysis.

### 3.2 Comparison of HRV features among the patient groups

We statistically compared the HRV features measured during the stress and relaxation tasks among the MDD, PD, and HC groups (**Supplementary Tables 3**, **4**). Significant differences were observed among the three groups in 13 HRV features among the 20 considered. Of these, 10 features—SDNN, RMSSD, pNN50, TRI, TINN, SD1, SD2, ApEn, SampEn, and CorDim—exhibited a significant main effect of the group in both tasks and the MDD and PD groups generally had lower values compared with HCs. RRI during the relaxation task and LF/HF during the stress task had a significant main effect of the group; however, no significant result was observed in the *post-hoc* analysis. Additionally, α2 during the relaxation task had higher values in the PD group compared with the HC group. These results were consistent with the altered ANS observed in depressive and anxiety disorders, as demonstrated in previous studies (24–26).

### 3.3 HRV feature changes between the stress and relaxation tasks

We examined the differences in HRV features between the stress and relaxation tasks within each participant to investigate the autonomic response to these mental tasks. **Supplementary Table 5** outlines the changes in HRV features (ΔHRV) between the stress and relaxation tasks. Our findings revealed that in the MDD group, 10 HRV features exhibited significant differences between the two tasks, whereas in the PD and HC groups, 14 features exhibited significant differences. Seven features—RRI, logLF, LFnu, HFnu, ApEn, α1, and α2—exhibited significant differences between the two tasks in all the three groups. These results suggested that the two mental tasks induced distinct autonomic responses, which were effectively captured by HRV metrics.

Existing literature established that HRV features generally decreased with stress, while features associated with LF, such as logLF, LFnu, and LF/HF, increased with stress owing to the dominance of sympathetic activity on LF (17–19). Consistent with these previous results, the HRV features that displayed significant differences between the two mental tasks in this study exhibited lower values in the stress condition (negative ΔHRV values), whereas features related to LF were higher in the stress condition (positive ΔHRV values). However, some features demonstrated an opposite pattern, such as TRI and TINN in the MDD and PD groups, ApEn in all the three groups, SampEn in the MDD and HC groups, α1 in all the three groups, and CorDim in the PD group, which presented higher values in the stress condition (positive ΔHRV values).

### 3.4 Classification of stress and relaxation tasks using HRV features and differences in classification performance among the groups

A random forest algorithm was employed to classify stress and relaxation responses using HRV features. We used the 20 HRV features as input data. A 10-fold CV repeated 20 times was used to evaluate the performance of the classification, implementing a subject-wise split to avoid data leakage. **Table 2** shows the performance metrics for classifying the responses. The performance measures of the overall group were evaluated by counting all the groups together in the test dataset without distinguishing among the three groups. The accuracy of the overall group was 0.7, demonstrating that stress and relaxation responses could be distinguished using HRV features. In addition, we calculated the same performance metrics separately for the three groups in the test set. The HC group had the highest scores in all the five metrics, followed by the PD and MDD groups, except the recall. The accuracy was 0.73, 0.69, and 0.67 for the HC, PD, and MDD groups, respectively. For the recall, the HC group still had the highest value, followed by the MDD and PD groups. These results suggested that the distinction between stress and relaxation responses was relatively accurate in the HC group compared with the patient groups. Particularly, there was approximately a 0.05 difference in accuracy between the MDD and HC groups, which indicated that for patients who are depressed, distinguishing between stress and relaxation based on HRV was relatively challenging compared with the healthy population.

**Table 2:** Performance measures for classifying the stress and relaxation tasks.

Model	Group	Accuracy	F1	Recall	Precision	AUC
Combined data model						
Overall		0.6986 ± 0.0055	0.7002 ± 0.0060	0.7098 ± 0.0105	0.6972 ± 0.0062	0.7708 ± 0.0035
MDD		0.6703 ± 0.0107	0.6770 ± 0.0132	0.6914 ± 0.0237	0.6635 ± 0.0107	0.7452 ± 0.0068
PD		0.6872 ± 0.0144	0.6773 ± 0.0144	0.6565 ± 0.0158	0.6995 ± 0.0167	0.7537 ± 0.0085
HC		0.7255 ± 0.0099	0.7338 ± 0.0090	0.7565 ± 0.0129	0.7126 ± 0.0117	0.7943 ± 0.0056
Combined data model with undersampling						
Overall		0.6974 ± 0.0094	0.6931 ± 0.0110	0.6983 ± 0.0140	0.6966 ± 0.0107	0.7662 ± 0.0078
MDD		0.6645 ± 0.0125	0.6686 ± 0.0145	0.6771 ± 0.0212	0.6605 ± 0.0114	0.7366 ± 0.0068
PD		0.6914 ± 0.0131	0.6779 ± 0.0126	0.6459 ± 0.0148	0.7136 ± 0.0180	0.7531 ± 0.0096
HC		0.7352 ± 0.0118	0.7359 ± 0.0128	0.7720 ± 0.0194	0.7032 ± 0.0117	0.7994 ± 0.0074
Separate data models						
MDD		0.6440 ± 0.0161	0.6324 ± 0.0242	0.6402 ± 0.0321	0.6555 ± 0.0234	0.7288 ± 0.0158
PD		0.6824 ± 0.0100	0.6819 ± 0.0139	0.6952 ± 0.0224	0.6977 ± 0.0167	0.7547 ± 0.0101
HC		0.7103 ± 0.0097	0.7092 ± 0.0103	0.7229 ± 0.0142	0.7177 ± 0.0116	0.7910 ± 0.0078

For the combined data model, the metrics were calculated separately for each patient group in the test dataset, in addition to the overall evaluation based on the entire test data. Separate data models were trained and tested, each using the data from one specific patient group exclusively. Results are presented as mean and standard deviation calculated from 20 repeats.

AUC, area under the curve; MDD, major depressive disorder; PD, panic disorder; HC, healthy control.

Sample sizes for the MDD and PD groups were 362 and 382, respectively, which were smaller compared with the HC group’s sample size of 556. We applied undersampling to the dataset and performed the classification again to investigate whether the relatively lower accuracy in the patient groups was owing to the difference in sample sizes during the training process. Using random undersampling, the sample sizes for the PD and HC groups were reduced to match the smallest sample size of 362. Starting with 362 samples for each group, the data was split into training and test datasets for classification, and performance was calculated.

We determined that even with undersampling applied to ensure an equal number of samples for training, the order of performance metrics remained unchanged among the groups, except for the precision (**Table 2**). For the precision, the PD group had the highest value, followed by HC and MDD groups. Accuracy for the HC and PD groups increased slightly with undersampling, whereas the MDD group exhibited a slight decrease. Overall accuracy based on the entire groups before and after applying undersampling remained nearly unchanged. This result suggested that the relatively higher accuracy in the HC group was not due to differences in sample sizes.

To further analyze the performance differences among the three groups, we built models exclusively trained and tested on the data from one group. The entire dataset was divided into three separate datasets for the MDD, PD, and HC groups. Subsequently, three separate models were trained and tested, each exclusively used the data from one specific group, which ensured that data from different groups did not interact during the training (**Table 2**). The HC group had the highest scores in all the five metrics, followed by the PD and MDD groups. The MDD and HC groups’ performance metrics decreased compared with those evaluated from the combined data model, whereas the PD group’s performance metrics remained similar to the combined data model. This could be owing to the decrease in the number of samples, and referencing data from other groups could have been helpful in training the model. These outcomes suggested that the reduced performance in the patient groups was intrinsic to the characteristics of the data.

### 3.5 Feature importance based on SHAP

**Figure 3:**
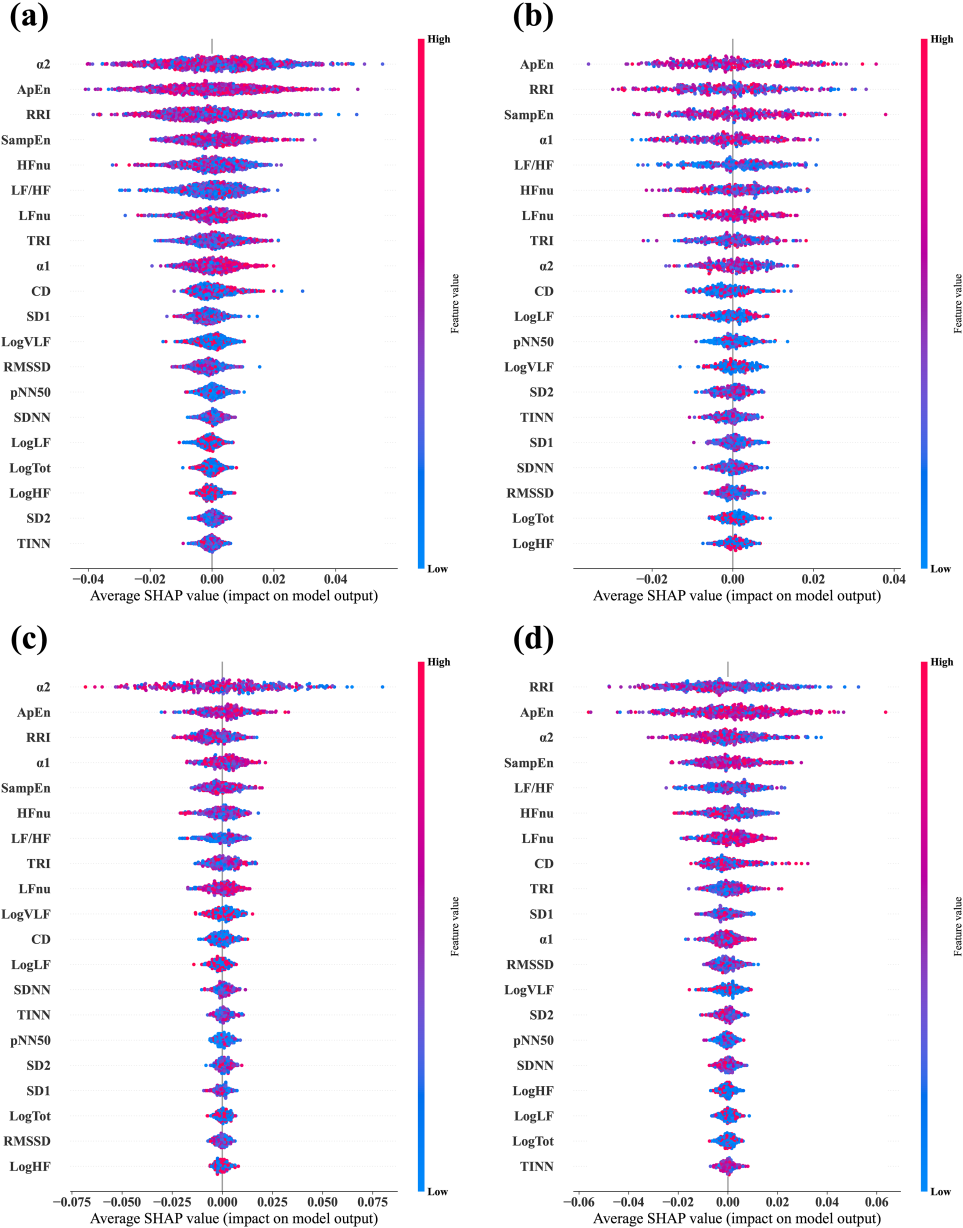
Average SHAP values evaluated from the four different classifier models. **(A)** Combined data model, which was trained and tested via data from all three groups—MDD, PD, and HC. **(B)** MDD-based model, which was trained and tested via data from the MDD group exclusively. **(C)** PD-based model, which was trained and tested via data from the PD group exclusively. **(D)** HC-based model, which was trained and tested via data from the HC group exclusively. In each plot, the features are arranged in descending order of importance.

We calculated the SHAP values via test datasets to identify the features critically responsible for the classification between stress and relaxation responses (**Figure 3**). SHAP values were calculated for four models: a combined data model that used data from all the three groups and three separate models based on the data from one specific group exclusively (MDD, PD, and HC). The importance of all 20 features was listed in descending order from the top for each model. For the combined data and PD-based models, the three top-ranked features were α2, ApEn, and RRI. In the MDD-based model, the top three features were ApEn, RRI, and SampEn, while in the HC group, the most significant features were RRI, ApEn, and α2. RRI and ApEn were consistently included in the top three features for all the models, which indicated their critical role in classification, although there were slight variations in their order of importance across the four models. Besides these two features, α2 and SampEn were included in the top three. Notably, α2 demonstrated dominant importance in the PD group compared with the other HRV features.

### 3.6 Group comparisons of ΔHRV

**Figure 4:**
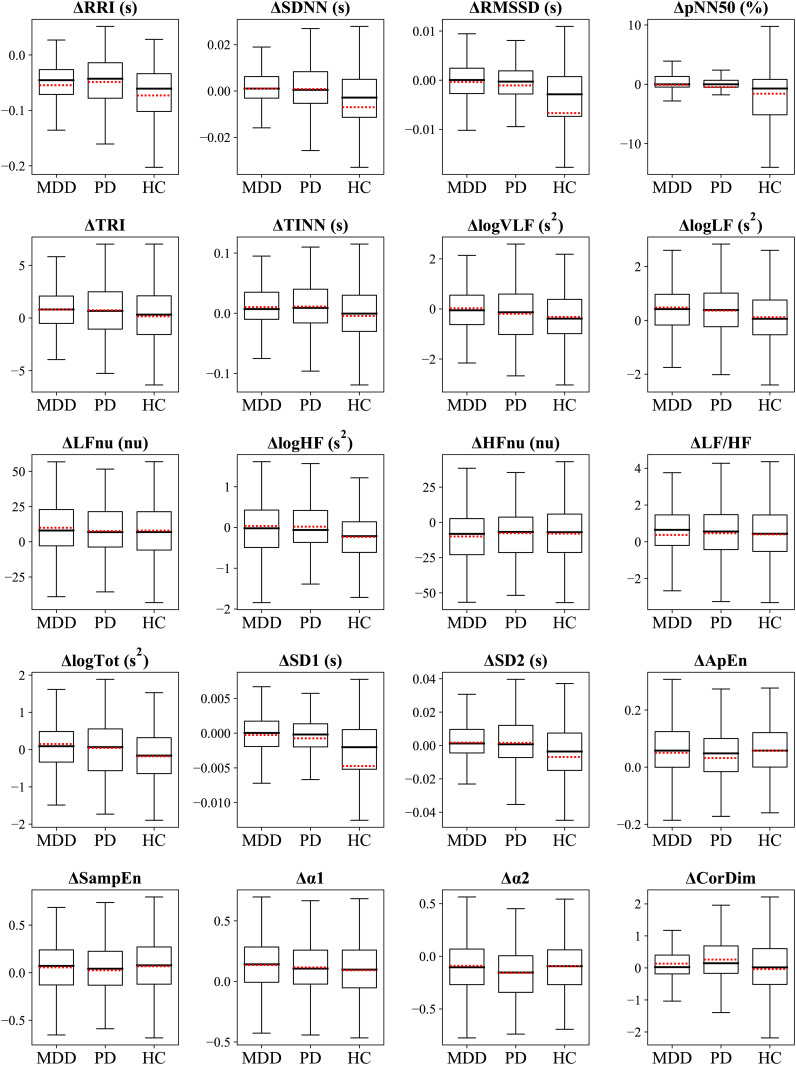
Box plots display the ΔHRV. Red dotted lines indicate mean values.

The ΔHRV represented the difference between relaxation and stress tasks, which was calculated to evaluate the participants’ autonomic reactivity. We hypothesized that the group with higher accuracy would exhibit greater reactivity, that is, absolute ΔHRV, compared with the other groups. This was as larger differences in feature values between the two tasks would make the classification process easier. We observed differences in the classification performance among the groups of MDD, PD, and HC. Therefore, we statistically compared ΔHRV values among the groups to examine whether psychiatric disorders affected the reactivity of the ANS to mental tasks. **Figure 4** illustrates the differences in ΔHRV among the groups, where the box plots illustrate the extent of HRV changes between the two tasks with red dotted lines denote the mean values.

A significant main effect of the group was observed in 11 features (**Table 3**). Specifically, HCs had significantly greater absolute changes than the MDD and PD groups in RRI, logHF, logTot, and SD2 and greater changes than the MDD group in logVLF. Conversely, HC had smaller absolute changes than the MDD and PD groups in TINN and logLF, while MDD had greater changes than HCs in TRI. The PD group exhibited significantly greater changes than the MDD and HC groups in α2 and greater changes than HCs in CorDim.

**Table 3:** Comparison of ΔHRV among the MDD, PD, and HC groups.

Feature (STR – RLX)	*F* (*P* value)	*η* ^2^	*Post-hoc P* value (Cohen’s *d*)			Absolute change
			MDD vs. PD	MDD vs. HC	PD vs. HC	
ΔRRI^a^ (s)	14.047 (<.001)	0.044	.452 (*d* = 0.125)	*<.001* (*d* = 0.365)	*<.001* (*d* = 0.466)	HC > MDD, PD
ΔSDNN (ms)	3.017 (.050)	0.009	1.00 (*d* = 0.022)	.117 (*d* = 0.184)	.124 (*d* = 0.178)	
ΔRMSSD (ms)	1.762 (.173)	0.005	ns			
ΔpNN50^a^ (%)	2.696 (.069)	0.009	ns			
ΔTRI^a^	3.301 (.038)	0.011	.970 (*d* = 0.024)	*.042* (*d* = 0.222)	.105 (*d* = 0.188)	MDD > HC
ΔTINN^a^ (ms)	4.185 (.016)	0.014	.982 (*d* = 0.019)	*.027* (*d* = 0.234)	*.024* (*d* = 0.239)	MDD, PD > HC
ΔlogVLF (s^2^)	4.326 (.014)	0.013	.254 (*d* = 0.169)	*.010* (*d* = 0.263)	.839 (*d* = 0.111)	HC > MDD
ΔlogLF (s^2^)	7.010 (<.001)	0.021	.886 (*d* = 0.098)	*.001* (*d* = 0.314)	*.041* (*d* = 0.266)	MDD, PD > HC
ΔLFnu (nu)	0.825 (.439)	0.003	ns			
ΔlogHF (s2)	7.607 (<.001)	0.023	1.00 (*d* = 0.017)	*.003* (*d* = 0.276)	*.005* (*d* = 0.396)	HC > MDD, PD
ΔHFnu (nu)	0.838 (.433)	0.003	ns			
ΔLF/HF	0.080 (.924)	0.000	ns			
ΔlogTot (s^2^)	7.383 (<.001)	0.022	.827 (*d* = 0.103)	*<.001* (*d* = 0.318)	*.036* (*d* = 0.276)	HC > MDD, PD
ΔSD1 (ms)	1.762 (.173)	0.005	ns			
ΔSD2 (ms)^a^	4.499 (.012)	0.017	.994 (*d* = 0.011)	*.010* (*d* = 0.255)	*.013* (*d* = 0.247)	HC > MDD, PD
ΔApEn	2.331 (.098)	0.007	ns			
ΔSampEn	1.164 (.313)	0.004	ns			
Δα1	1.907 (.149)	0.006	ns			
Δα2	4.239 (.015)	0.013	*.035* (*d* = 0.258)	1.00 (*d* = 0.020)	*.031* (*d* = 0.251)	PD > MDD, HC
ΔCorDim^a^	4.147 (.016)	0.013	.494 (*d* = 0.118)	.240 (*d* = 0.153)	*.012* (*d* = 0.266)	PD > HC

*Post-hoc P* values in italics <.05.

ns, no significant main effect; MDD, major depressive disorder; PD, panic disorder; HC, healthy control.

a Welch’s one-way ANOVA and Games-Howell *post-hoc* analysis were used. Except for these cases, Fisher’s one-way ANOVA and Bonferroni *post-hoc* analysis were employed.

The HC group demonstrated greater absolute changes than the MDD group in five features, while the MDD group exhibited larger absolute changes than the HC group in three features. Comparison of the HC and PD groups revealed greater absolute changes than the other in four features. A significant difference between the MDD and PD groups was observed only in α2, and the PD group had a greater absolute change than the MDD group.

### 3.7 Personalized longitudinal scaling of the HRV features

Participants were measured multiple times over an extended period, which provided an opportunity to collect data at various time points for each individual. We normalized the data for each individual over the time axis to leverage this benefit (**Figure 2**). We performed classification based on the scaled HRV data via the same methods to determine whether this personalized longitudinal scaling improved the classification between stress and relaxation responses.

Initially, we aimed to understand the impact of scaling on the data using t-SNE for visualization to determine if the separation between stress and relaxation became more distinct after scaling (**Supplementary Figure 3**). The t-SNE visualization of the HRV data before and after longitudinal scaling illustrated the improved separation of stress and relaxation classes post-scaling, which suggested an improvement in classification performance.

### 3.8 Scaled HRV feature changes between the stress and relaxation tasks

We examined the differences in the longitudinally scaled HRV features the between stress and relaxation tasks within each participant (**Supplementary Table 6**). We determined that 14, 12, and 16 HRV features exhibited significant differences between the two tasks in the MDD, PD, and HC groups, respectively. Furthermore, seven features—RRI, LFnu, HFnu, LF/HF, ApEn, α1, and α2—exhibited significant differences between the two tasks in all the three groups. The MDD and HC groups exhibited a higher number of significantly different HRV features after scaling, whereas the PD group exhibited a decreased number of significantly different HRV features post-scaling.

Furthermore, similar to the unscaled HRV features, the scaled HRV features that demonstrated significant differences between the two mental tasks exhibited lower values in the stress condition (negative ΔHRV_scaled_ values), whereas features related to LF were higher in the stress condition (positive ΔHRV_scaled_ values). However, the following features presented higher values in the stress condition (positive ΔHRV_scaled_ values): SDNN in the MDD group, TRI and TINN in the MDD and PD groups, SD2 in the MDD group, ApEn in all the three groups, SampEn in the MDD and HC groups, α1 in all the three groups, and CorDim in the MDD and PD groups.

### 3.9 Classification of the stress and relaxation tasks using scaled HRV features

We performed classification via scaled HRV data and followed the same methodology as with the unscaled data (**Table 4**). The overall accuracy increased significantly from 0.70 with unscaled data to 0.94 after scaling. When we examined the individual metrics for MDD, PD, and HC groups, the HC group demonstrated the highest values across all the metrics, followed by the MDD and PD groups, except for the precision. Accuracy was 0.94, 0.90, and 0.96 for the MDD, PD, and HC groups, respectively. These findings demonstrated differences in classification performance across the groups, and the HC group achieved the highest accuracy compared with the other two disease groups. Notably, with unscaled data, the accuracy of the PD group was slightly higher than that of the MDD group. However, after scaling, the MDD group exhibited higher accuracy than the PD group.

**Table 4:** Performance measures for classifying stress and relaxation tasks based on the longitudinally scaled HRV data.

Model	Group	Accuracy	F1	Recall	Precision	AUC
Combined data model						
Overall		0.9391 ± 0.0021	0.9389 ± 0.0022	0.9399 ± 0.0034	0.9389 ± 0.0030	0.9798 ± 0.0015
MDD		0.9420 ± 0.0036	0.9413 ± 0.0036	0.9301 ± 0.0041	0.9528 ± 0.0056	0.9799 ± 0.0011
PD		0.9048 ± 0.0041	0.9044 ± 0.0043	0.9005 ± 0.0072	0.9084 ± 0.0042	0.9610 ± 0.0027
HC		0.9603 ± 0.0025	0.9608 ± 0.0025	0.9732 ± 0.0041	0.9486 ± 0.0037	0.9916 ± 0.0013
Separate data models						
MDD		0.9350 ± 0.0051	0.9336 ± 0.0053	0.9291 ± 0.0079	0.9413 ± 0.0067	0.9801 ± 0.0031
PD		0.9057 ± 0.0086	0.9065 ± 0.0094	0.9238 ± 0.0104	0.8935 ± 0.0103	0.9542 ± 0.0052
HC		0.9545 ± 0.0038	0.9543 ± 0.0039	0.9572 ± 0.0044	0.9529 ± 0.0045	0.9881 ± 0.0015

For the combined data model, the metrics were calculated separately for each patient group in the test dataset, in addition to the overall evaluation based on the entire test data. Separate data models were trained and tested, each using the data from one specific patient group exclusively. Results are presented as mean and standard deviation calculated from 20 repeats.

AUC, area under the curve; MDD, major depressive disorder; PD, panic disorder; HC, healthy control.

Furthermore, we compared the three separate models, each utilizing the data from one specific group exclusively, which was similar to our approach with unscaled data (**Table 4**). All the three models demonstrated a significant improvement in classification performance after scaling. Best classification results were observed for the HC group, followed by the MDD and PD groups, respectively. These results suggested the substantial impact of personalized longitudinal scaling on our classification models’ performance across different groups.

### 3.10 Feature importance after longitudinal scaling

**Figure 5:**
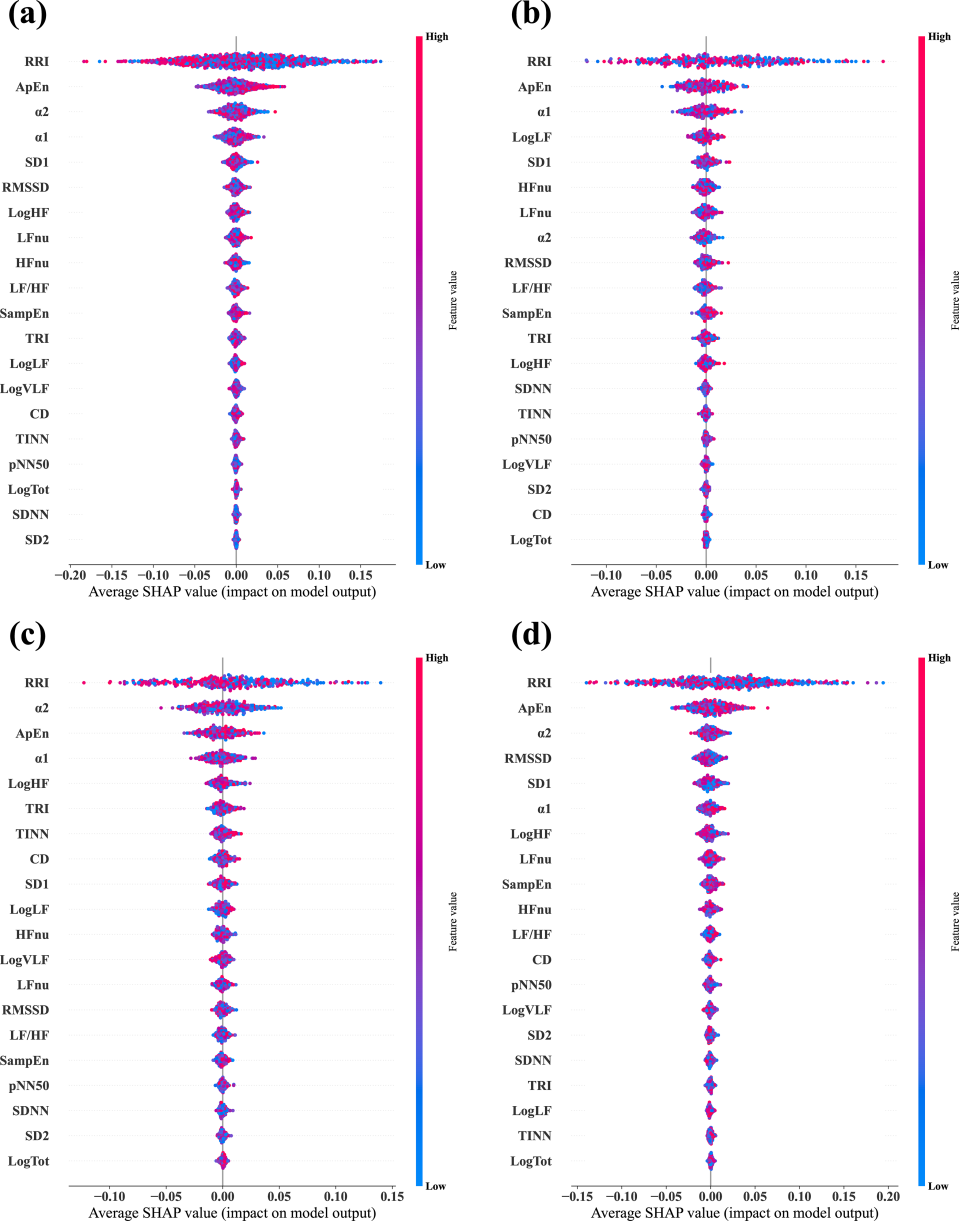
Average SHAP values evaluated from the four different classifier models via the longitudinally scaled HRV data. **(A)** Combined data model, which was trained and tested via data from all three groups, —MDD, PD, and HC. **(B)** MDD-based model, which was trained and tested via data from the MDD group exclusively. **(C)** PD-based model, which was trained and tested via data from the PD group exclusively. **(D)** HC-based model, which was trained and tested via data from the HC group exclusively. In each plot, the features are arranged in descending order of importance.

We applied the same methodology used for the unscaled data to calculate SHAP values for the classification based on scaled data (**Figure 5**). The key finding was that RRI emerged as the most important feature across all the models. When the top three features were considered, only the order changed in the combined data and PD models. In the MDD group, SampEn was replaced by α1, while in the HC group, the composition and order of the top three features remained unchanged. RRI and ApEn consistently ranked as essential features across all the groups, which was consistent with the results from the unscaled data.

### 3.11 Group comparisons of the scaled ΔHRV

**Figure 6:**
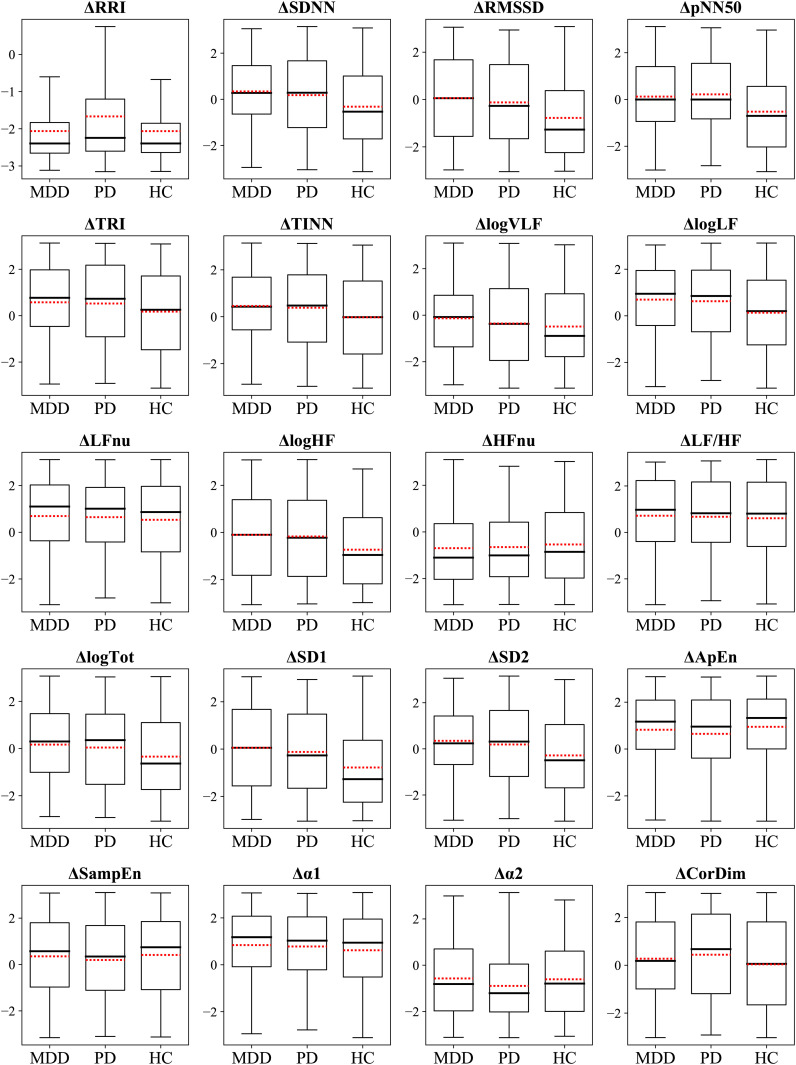
Box plots display the ΔHRV_scaled_. Red dotted lines indicate mean values.

**Figure 6** illustrates the differences in scaled ΔHRV among the groups. We compared scaled ΔHRV values among the MDD, PD, and HC groups (**Table 5**) and found a significant main effect of the group in 11 HRV features. HC participants exhibited greater absolute changes between the stress and relaxation tasks than the MDD and PD groups in seven features: SDNN, RMSSD, pNN50, logHF, logTot, SD1, and SD2. Conversely, the HC group had smaller changes in TINN and logLF than the MDD and PD groups. In RRI, the MDD and HC groups had greater changes than the PD group. The MDD group also demonstrated a greater change in TRI than the HC group.

**Table 5:** Comparison of ΔHRV_scaled_ among the MDD, PD, and HC groups.

Feature (a.u.) (STR – RLX)	*F* (*P* value)	*η* ^2^	*Post-hoc P* value (Cohen’s *d*)			Absolute change
			MDD vs. PD	MDD vs. HC	PD vs. HC	
ΔRRI^a^	6.503 (0.002)	0.027	*0.004* (*d* = 0.331)	1.000 (*d* = 0.001)	*0.002* (*d* = 0.335)	HC, MDD > PD
ΔSDNN^a^	10.500 (< 0.001)	0.031	0.594 (*d* = 0.101)	*< 0.001* (*d* = 0.412)	*0.006* (*d* = 0.293)	HC > MDD, PD
ΔRMSSD	14.496 (< 0.001)	0.043	0.986 (*d* = 0.099)	*< 0.001* (*d* = 0.467)	*< 0.001* (*d* = 0.377)	HC > MDD, PD
ΔpNN50	14.463 (< 0.001)	0.043	1.000 (*d* = 0.060)	*< 0.001* (*d* = 0.391)	*< 0.001* (*d* = 0.453)	HC > MDD, PD
ΔTRI^a^	3.834 (0.022)	0.012	0.953 (*d* = 0.030)	*0.032* (*d* = 0.238)	0.089 (*d* = 0.198)	MDD > HC
ΔTINN	5.698 (0.004)	0.017	1.000 (*d* = 0.045)	*0.008* (*d* = 0.291)	*0.029* (*d* = 0.240)	MDD, PD > HC
ΔlogVLF	2.541 (0.080)	0.008	ns			
ΔlogLF	8.594 (< 0.001)	0.026	1.000 (*d* = 0.045)	*< 0.001* (*d* = 0.350)	*0.004* (*d* = 0.303)	MDD, PD > HC
ΔLFnu	0.585 (0.557)	0.002	ns			
ΔlogHF^a^	9.541 (< 0.001)	0.028	0.924 (*d* = 0.039)	*< 0.001* (*d* = 0.362)	*0.002* (*d* = 0.325)	HC > MDD, PD
ΔHFnu	0.585 (0.557)	0.002	ns			
ΔLF/HF	0.258 (0.773)	0.001	ns			
ΔlogTot	6.038 (0.003)	0.018	1.000 (*d* = 0.076)	*0.004* (*d* = 0.314)	*0.041* (*d* = 0.228)	HC > MDD, PD
ΔSD1	14.505 (< 0.001)	0.043	0.985 (*d* = 0.100)	*< 0.001* (*d* = 0.467)	*< 0.001* (*d* = 0.377)	HC > MDD, PD
ΔSD2^a^	9.693 (< 0.001)	0.028	0.613 (*d* = 0.098)	*< 0.001* (*d* = 0.397)	*0.009* (*d* = 0.280)	HC > MDD, PD
ΔApEn	2.043 (0.130)	0.006	ns			
ΔSampEn	0.932 (0.394)	0.003	ns			
Δα1	1.214 (0.298)	0.004	ns			
Δα2^a^	2.670 (0.070)	0.008	ns			
ΔCorDim^a^	2.800 (0.062)	0.009	ns			

*Post-hoc P* values in italics <.05.

ns, no significant main effect; MDD, major depressive disorder; PD, panic disorder; HC, healthy control.

a Welch’s one-way ANOVA and Games-Howell *post-hoc* analysis were used. Except for these cases, Fisher’s one-way ANOVA and Bonferroni *post-hoc* analysis were employed.

The HC group had greater absolute changes than the MDD group in seven features, whereas the MDD group exhibited larger absolute changes than the HC group in three features. The HC group had greater absolute changes than the PD group in eight features, whereas the PD group exhibited larger absolute changes than the HC group in two features. A significant difference between the MDD and PD groups was observed only in RRI, and the MDD group exhibited a greater absolute change than the PD group.

### 3.12 Classification using an MLP algorithm

To verify whether our findings were influenced by the choice of the machine-learning algorithm, we utilized the MLP algorithm to conduct the same classification tasks previously conducted via the random forest algorithm. Furthermore, we applied the same classification to the longitudinally scaled HRV data using MLP classifiers.

Results obtained from the MLP models were consistent with those generated by the random forest algorithm (**Supplementary Table 7**). Before we applied the personalized longitudinal scaling, the order of accuracy was HC, PD, and MDD in the MLP model. After its application, the accuracy increased to over 0.9, and the order of accuracy was HC, MDD, and PD in the MLP model. This consistency with the random forest algorithm results indicated that our findings were not affected by the choice of the machine-learning algorithm; rather, they stemmed from the inherent characteristics of the data itself.

## 4 Discussion

**Figure 7:**
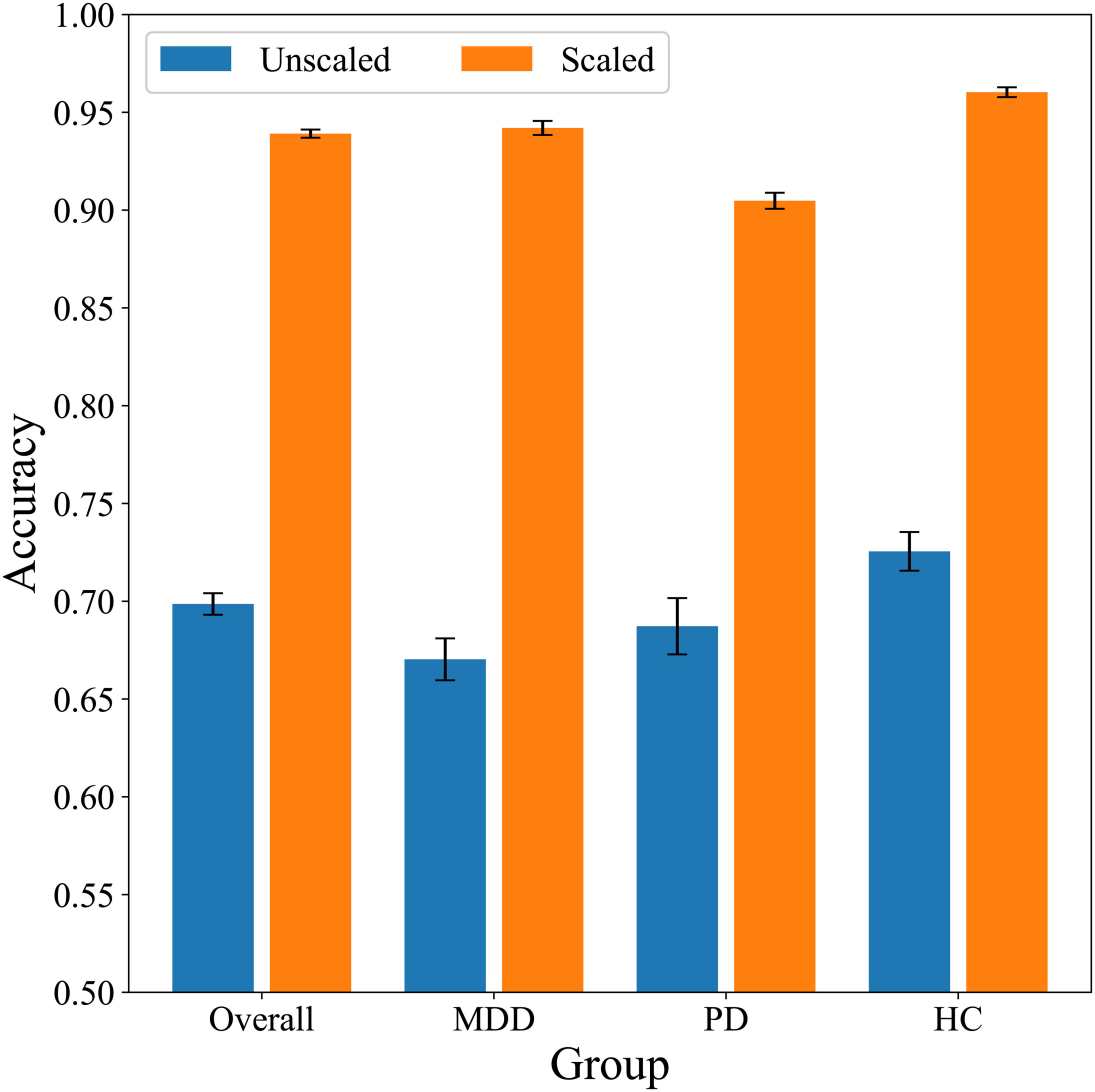
Summary of classification accuracies based on combined data models (**Tables 2**, **4**).

We differentiated stress and relaxation based on HRV features in groups with MDD, PD, and HCs via a random forest algorithm. Classification accuracies for the MDD, PD, and HC groups were 0.67, 0.69, and 0.73, respectively, which indicated that the classification of stress and relaxation was more accurate for healthy individuals compared with patients with MDD and PD (**Figure 7**). A personalized longitudinal scaling of HRV data improved the accuracies for all the groups, and the MDD, PD, and HC groups reached accuracies of 0.94, 0.90, and 0.96, respectively (**Figure 7**). This suggested the potential of personalized scaling in monitoring the condition of patients with psychiatric disorders. Results obtained from the MLP models were consistent with those generated by the random forest classifier, which suggested that our findings were not dependent on the specific algorithm used.

Patients’ HRV values were significantly lower than those of the healthy individuals (**Supplementary Table 3**). We noticed significant differences among the groups in 13 HRV features. The MDD and PD groups displayed lower values compared with the HC group. These findings aligned with previous research, which indicated that individuals with depression and PD had reduced HRV. This suggested lower autonomic flexibility and higher sympathetic dominance (24–26).

Several features demonstrated significant differences between the stress and relaxation tasks, which indicated that HRV effectively distinguished between these states (**Supplementary Table 5**). Particularly, seven features—RRI, logLF, LFnu, HFnu, ApEn, α1, and α2—exhibited significant differences between the two tasks in all three groups. Among these, RRI, ApEn, and α2 were also identified as highly important features based on SHAP evaluation (**Figure 3**), which suggested their potential importance in classification. Moreover, the PD and HC groups had more features that depicted significant differences between the tasks compared with the MDD group. This indicated potential challenges for the MDD group in distinguishing between stress and relaxation based on HRV features.

Previous research determined that most HRV features decreased under stress owing to increased sympathetic and decreased parasympathetic activity (17–19, 71, 72). In contrast, LF-related features, closely linked to sympathetic activity, tended to increase under stress (17–19, 71, 72). Our results also indicated that most features with statistically significant differences between the two tasks displayed lower values during stress, whereas LF-related features increased during stress. However, exceptions were observed for geometric features, such as TRI and TINN, and nonlinear features, such as ApEn, SampEn, α1, and CorDim, which presented lower HRV values during the relaxation task. Methodological variations existed across studies, which included differences in stimulation methods, order of stimuli, and relaxation techniques (19). For instance, α1 decreased under physical stress but increase under psychological stress (73, 74). Therefore, HRV responses to a specific stress stimulus may not consistently exhibit lower values across all features. These findings emphasized the complexity of HRV responses and the importance of considering multiple factors when interpreting HRV data in the context of stress and relaxation (19). Future studies should conduct additional tests to investigate lower HRV during the relaxation task compared with the stress task.

Our most significant finding was that under the same experimental conditions, distinguishing stress and relaxation in the PD and MDD groups compared with the HC group was relatively more challenging. With unscaled HRV, the accuracy for the HC group was 0.73, whereas it was relatively lower for the PD and MDD groups at 0.69 and 0.67, respectively (**Table 2**). We analyzed the reasons for this difference via various methods. First, we matched the sample size through random undersampling since the HC group had the highest number of samples; however, the order of the performance metrics remained similar among the groups (**Table 2**). Second, we built classifier models exclusively for each group to ensure that data from different groups did not interact during the training process. However, the accuracy for the HC group remained higher than that of the other two patient groups (**Table 2**). These findings suggested that the diminished performance was inherent to the traits of the PD and MDD groups.

In addition, we evaluated the important features used in classifier models with SHAP (**Figure 3**). Although the overall ranking of the 20 features varied across the four models. ApEn and RRI were consistently among the top three features in all four models. Additionally, SampEn and α2 were included as important features. This indicated that despite group differences, the key indicators used for stress-relaxation classification based on the random forest presented no substantial differences across the models. Similar results were observed in within-subject comparison statistics (**Supplementary Table 5**), where RRI, ApEn, and α2 exhibited distinct differences between the stress and relaxation states across all the groups. Combining the results from SHAP and statistical analysis, we can infer that RRI, ApEn, and α2 are expected to play a crucial role in classification.

To further investigate the difference in classification accuracy among the groups, we compared their ΔHRV values (**Table 3**). ΔHRV was calculated to assess participants’ autonomic reactivity. Our previous works demonstrated that patients with psychiatric disorders had pathologically altered autonomic responses compared with healthy individuals, which resulted in different reactions to mental tasks (44, 75–77). A larger absolute ΔHRV indicated a more distinct ANS response to the two tasks, which suggested better classification performance for groups with greater reactivity. We hypothesized that the higher-accuracy group would show greater reactivity, that is, larger absolute ΔHRV, than the other groups.

Analysis based on unscaled ΔHRV demonstrated that 11 features had significant differences among groups (**Table 3**). Among these, four features—RRI, logHF, logTot, and SD2—had larger absolute ΔHRV values in the HC group compared with the two patient groups. RRI, identified as a highly important indicator by SHAP, likely significantly contributed to the higher classification accuracy in the HC group (**Figure 3**). Furthermore, in the HC-based classifier model that used only HC data, RRI was the most important feature, underscoring its importance (**Figure 3**). Conversely, for α2, another important feature indicated by SHAP, the PD group exhibited greater absolute changes than the MDD and HC groups. TINN and logLF presented greater absolute changes in the two patient groups compared with the HC group.

In comparing the HC group to the MDD group, more features with greater absolute changes, including the important feature RRI, were observed in the HC group. Although the MDD group had some features with greater absolute changes than the HC group, these features were less important than RRI. These results suggested that the HC group likely achieved better classification results because they exhibited higher reactivity in both a greater number of features and more important features compared with the MDD group. We compared HC and PD groups and observed that each group had greater absolute changes in four features compared with the other. Interestingly, the PD group displayed greater absolute changes in an important feature, α2. This suggested that a comparison based on the number of highly reactive features and inclusion of important features may not be sufficient to clearly explain why the HC group outperformed the PD group regarding classification. The superiority of HRV features in the HC group over those in the PD group for classification was demonstrated through various methods in our study. We plan to further explore this reason comprehensively. Thus, the HC group exhibited significantly greater ΔHRV in a larger number of or more important features than the patient groups, indicating more pronounced autonomic reactivity. This enhanced reactivity likely contributes to the higher classification accuracy observed in the HC group.

Our participants made up to five visits over a period of 12 weeks and completed five tasks during each visit. Data collected from these visits and tasks were used for personalized scaling. Our goal was to gather as much data as possible while exposing the participants to various experimental conditions. Our study demonstrated that personalized longitudinal scaling significantly improved classification performance across all the participant groups. We utilized t-SNE visualization and observed improved separation of feature values into stress and relaxation states (**Supplementary Figure 3**). Individual HRV responses varied across multiple visits, which reflected the influence of both stimuli and daily state of each participant, as depicted in **Figure 2** (78, 79). Changes in HRV may be more strongly influenced by daily states than by specific external stimuli. If this variability in HRV is not adequately normalized, classifying tasks based on HRV data can pose significant challenges. In cases where a substantial amount of individual data is accumulated in the long term, time-axis scaling (i.e., longitudinal scaling) can be applied, which can help reduce variability occurring at each time point (e.g., a visit in this study). Therefore, longitudinal scaling can lead to a clearer separation between HRV values measured during different tasks.

We conducted the analysis on the scaled HRV data via the same method that was applied to the unscaled data to understand classification performance based on the scaled data. Seven scaled features—RRI, LFnu, HFnu, LF/HF, ApEn, α1, and α2—exhibited significant differences between the stress and relaxation tasks in all the three groups (**Supplementary Table 6**). Among these, RRI, ApEn, α1, and α2 were also identified as highly important features based on the SHAP evaluation (**Figure 5**), which suggested their potential importance in classification. The HC group had four more features that exhibited significant differences between the two tasks compared with the PD group, which suggested a relative difficulty for the PD group in classification between stress and relaxation.

Personalized scaling dramatically improved classification performance in all the three groups (**Table 4**). The accuracy exceeded 0.9, which allowed for precise differentiation between stress and relaxation states. These outcomes demonstrated the potential of personalized data scaling to monitor individual patient conditions with high accuracy. Interestingly, the accuracy remained highest in the HC group, followed by the MDD and PD groups. Before the scaling, the order of accuracy was HC, PD, and MDD; however, after the scaling, the order of accuracy for PD and MDD reversed.

The SHAP analysis on the scaled HRV data revealed that RRI was the most important feature across all classification models (**Figure 5**). When compared with other features, RRI’s importance was dominantly higher. Although RRI was already one of the top three important features in the unscaled HRV data, its importance increased significantly after scaling, as demonstrated by comparing the two in **Figures 3**, **5**. This indicated that the role of RRI in the classification became significantly more crucial post-scaling. In the scaled HRV data, ApEn and α2 were also included in the top three important features, similar to the unscaled data.

The RRI is a sensitive indicator of changes in both the sympathetic and parasympathetic nervous systems, making it valuable for detecting autonomic variations under conditions of stress (80–82). Previous studies have identified RRI or its inverse, average heart rate, as key indicators for stress classification (80–82). Furthermore, RRI’s high temporal resolution enhances its effectiveness in stress detection, allowing for accurate classification even with short measurement periods of 30 seconds (81, 82). Additionally, entropy-based measures such as ApEn capture heartbeat irregularity, which typically decreases under stress, thereby making it an effective feature for stress detection (54, 83, 84). ApEn has been used to classify stressful events, underscoring its relevance as a stress indicator (54). The DFA α2 reflects the complexity and fractal characteristics of long-term HRV, capturing self-similarity across time scales and changes in adaptability under stress (17, 53, 58). Prior studies have demonstrated that stress-induced shifts in the ANS toward sympathetic dominance alter HRV complexity, resulting in changes to α2 (85, 86). These findings underscore the utility of α2 for assessing cardiac autonomic regulation across various conditions. Although stimuli used to induce stress or relaxation in studies vary, complicating direct comparisons, our findings align with previous research where RRI and nonlinear HRV measures are identified as significant features for stress classification.

We compared the scaled ΔHRV values among the different groups to investigate differences in classification performance via the same method as the unscaled data (**Table 5**). We determined that the absolute change of RRI, the most important feature, was greater in the MDD and HC groups compared with the PD group. This suggested that the MDD and HC groups could have had an advantage in classification compared with the PD group. Additionally, we noticed that the HC group exhibited greater absolute changes in more features than the MDD group, which suggested a relative advantage in classification for the HC group. Overall, our findings suggested that a group that displayed a greater number of features with higher reactivity tended to exhibit better performance compared with other groups when the scaled HRV data was used for classification.

Recent studies on monitoring technologies for patients with psychiatric disorders focused on obtaining longitudinal data, such as ecological momentary assessment and physiological data, to observe patient conditions and use the findings to improve treatment (87, 88). Our results indicated that personalized data scaling could enhance the accuracy of assessing patient conditions in studies that monitored patients via physiological signals. Although personalized scaling requires a substantial amount of accumulated data for each individual, it is expected to significantly improve classification performance.

In the recent times, artificial intelligence techniques are increasingly being used in research to detect stress based on HRV (21). Such studies employ diverse methods, from classical rule-based techniques such as fuzzy logic to classical machine learning approaches including support vector machine, random forest, and k-nearest neighbors. Advanced methods, including deep learning and hybrid approaches combining classical algorithms with neural networks, further demonstrate the variety of stress detection techniques that are used in studies (21, 89). Many studies have investigated beyond just HRV, leveraging multimodal sensor data. Signals such as ECG, photoplethysmogram (PPG), electrodermal activity (EDA), electromyogram, or respiration are simultaneously measured and utilized (21). Whether using HRV or multimodal sensors, most studies report classification accuracies between 70% and 99% (21). For example, one study achieved 75% accuracy using HRV and a random forest algorithm (90), while another reached 90% with an artificial neural network and HRV (91). Our results, with HRV-based random forest and MLP models achieving 70% to over 90% accuracy, align with these prior findings.

However, most studies focus on non-clinical populations, and analysis of stress detection for individuals with psychiatric conditions is lacking (21). Private datasets used in previous studies primarily focus on healthy individuals; furthermore, publicly available datasets such as SWELL-KW and PhysioNet’s driving database also mainly target healthy individuals (21, 90, 91). However, studies addressing stress detection in psychiatric population groups, such as those with MDD or PD, are uncommon.

Our study addresses this gap by conducting comparative experiments with clinical populations (MDD and PD) and HCs under controlled stress-relaxation protocols. The observed differences in stress-relaxation classification highlight the need to consider disparities between patients and healthy individuals when developing ANS monitoring technologies. These findings emphasize the importance of tailored solutions for both clinical and everyday settings, thereby addressing the unique autonomic characteristics of psychiatric populations.

Thus, the clinical implications of this study indicate that HRV has significant potential as a biomarker for stress, particularly in differentiating between stress and relaxation states across the three groups. Our findings suggest that effective stress monitoring should consider the varying autonomic responses of patient groups and healthy individuals to improve classification accuracy. Furthermore, the implementation of personalized data scaling significantly enhanced classification performance, indicating that individualized HRV-based monitoring could offer a more reliable and tailored stress assessments for managing psychiatric conditions.

### 4.1 Limitations

The number of participants measured by the experiment was small. Although the sample size exceeded 1000 owing to multiple individual visits, the number of participants per group was approximately 40–60. Particularly, the number of patients in the PD and MDD groups was smaller than that in the HC group. Recruiting more participants would enable further research into how this might impact classification accuracy between groups.

Importantly, our patient groups were on medication during the 12-week experiment, and we did not specifically analyze their potential impact on our results. Ongoing research into the effects of therapeutic drugs on HRV suggests varying impacts. Antidepressants have been linked to alterations in HRV; however, definitive evidence remains inconclusive. One meta-analysis reported that TCAs substantially reduced HRV, while other antidepressants showed minimal effects (92). In contrast, a large study involving more than 2000 participants found no association between HRV and MDD itself. However, MDD patients on SSRIs, SNRIs, and TCAs displayed a significantly reduced HRV (93). These findings imply that the antidepressants, rather than MDD alone, may explain the reduced HRV observed in the study participants, as all were undergoing treatment. Therefore, we cannot entirely eliminate the possibility that the observed differences in classification performance and HRV reactivity among the groups could be influenced by medication. Nevertheless, considering that patients are likely to be on medication in real-world applications, our findings remain relevant for practical therapeutic environments. Furthermore, this study did not distinguish between treatment responders and non-responders within the patient groups. In future, we intend to differentiate these response groups, enabling a more detailed analysis of differences in stress reactivity and stress-relaxation classification performance between responders and non-responders.

We utilized SHAP as a representative method to calculate feature importance and employed statistical analysis methods to obtain complementary data on HRV reactivity. Alternative calculation methods for feature importance, such as permutation feature importance and local interpretable model-agnostic explanations, exist (94, 95). These methods can be explored in future studies. The statistical test identifies features that differ significantly among groups, while SHAP values highlight features that most contribute to the model’s predictions and considered the full complexity of the data. Both statistical analysis and SHAP values play distinct yet complementary roles in indicating important metrics. Features that are both statistically significant and have high SHAP values might be considered as candidates for essential predictors.

We used only a basic MLP model. With recent advancements in deep learning, various neural network models have shown promising results in medical sciences, including psychiatry (96). The reason for applying MLP was to demonstrate that our results are not confined to a specific algorithm. We chose MLP to perform the same classification via an entirely different algorithm from random forest. For future studies, we aim to experiment with more advanced neural network architectures to enhance classification performance.

In this study, we focused exclusively on HRV as a measure of responses to stress and relaxation. Although there are several other physiological markers that can be employed to monitor stress responses, such as EDA, respiratory rate, blood pressure variability, and electroencephalography, HRV provides several advantages (97–100). For example, the development of wearable devices has made it more accessible for continuous monitoring of HRV. Moreover, HRV measurements are typically less susceptible to external noise and environmental factors, resulting in more reliable and stable outcomes. The EDA is one of the physiological signals measured by wearable devices, commonly used in stress research, and has demonstrated promising results. However, unlike HRV, which measures both sympathetic and parasympathetic activity, EDA can only measure sympathetic activity. Considering the limited research conducted on automated stress detection in psychiatric disorders, we prioritized HRV for its ability to provide a more comprehensive view of autonomic balance. In this study, HRV features were extracted from ECG signals. Use of commercial or research-grade wearable devices to measure PPG and derive HRV can help future research, enabling real-time stress monitoring for individuals with psychiatric conditions.

## 5 Conclusion

Our study utilized HRV features to distinguish stress and relaxation responses among groups with MDD and PD and HCs via a random forest algorithm. Classification accuracies were 0.67, 0.69, and 0.73 for the MDD, PD, and HC groups, respectively, which indicated higher accuracy in healthy individuals. Personalized longitudinal scaling of HRV data improved classification accuracies, and reached 0.94, 0.90, and 0.96 for the MDD, PD, and HC groups, respectively, which suggested the potential of personalized scaling in monitoring a patient’s conditions based on HRV measurements. Results produced by the MLP models were in line with those by the random forest classifier, which indicated that our findings were not reliant on a particular algorithm.

Our findings revealed that it was more challenging to differentiate stress and relaxation in the PD and MDD groups than in HCs, partly owing to the intrinsic characteristics of patient data that reflected altered autonomic responses. Additionally, the HC group demonstrated greater autonomic reactivity in a larger number of and more significant features, which potentially contributed to higher classification accuracy. These results underscore the potential of HRV metrics as biomarkers for stress and emphasize the importance of accounting for differences in autonomic responses between patients and healthy individuals when developing stress monitoring technologies in both clinical and everyday settings.

## Data Availability

The datasets presented in this article are not readily available because of privacy restrictions. Requests to access the datasets should be directed to david0203@korea.ac.kr.
