# Supplementary material for: Automated classification of stress and relaxation responses in major depressive disorder, panic disorder, and healthy participants via heart rate variability
Source: Front Psychiatry. 2025 Jan 9;15:1500310. doi: 10.3389/fpsyt.2024.1500310 (PMC11754969; doi:10.3389/fpsyt.2024.1500310)
Supplement: Supplementary file 1 [file DataSheet1.pdf]

Supplementary Material

1 Supplementary Tables

Supplementary Table 1. The 20 HRV features used in this study.

Features	Descriptions
**Time-domain**	
RRI (s)	Mean RR intervals
SDNN (s)	Standard deviation of RR intervals
RMSSD (s)	Root mean square of successive RR interval differences
pNN50 (%)	Percentage of successive RR intervals differing more than 50 ms
TRI	Integral of the histogram of the RR interval divided by its height
TINN (s)	Baseline width of the RR interval histogram
**Frequency-domain**	
logVLF (s^2)	Logarithm-transformed power in the VLF band (0−0.04 Hz)
logLF (s^2)	Logarithm-transformed power in the LF band (0.04−0.15 Hz)
LFnu (nu)	Relative power of the LF band
logHF (s^2)	Logarithm-transformed power in the HF band (0.15−0.4 Hz)
HFnu (nu)	Relative power of the HF band
LF/HF	Ratio between LF and HF band powers
logTot (s^2)	Logarithm-transformed total power
**Poincaré plot and nonlinear domain**	
SD1 (s)	Standard deviation of the Poincaré plot perpendicular to the line of identity
SD2 (s)	Standard deviation of the Poincaré plot along the line of identity
ApEn	Approximate entropy
SampEn	Sample entropy
α1	Short-term scaling exponent of DFA
α2	Long-term scaling exponent of DFA
CorDim	Correlation dimension

Supplementary Table 2. Post-hoc *P* values for comparisons among the three groups shown in Table 1.

Data	Post-hoc (P value)		
	MDD vs. PD	MDD vs. HC	PD vs. HC
HAMD[a]	.062	< .001	< .001
HAMA[a]	0.701	< .001	< .001
PDSS[a]	< .001	< .001	< .001

MDD: major depressive disorder; PD: panic disorder; HC: healthy control.

[a]Welch's one-way ANOVA and Games-Howell post-hoc analysis

Supplementary Table 3. Comparison of HRV features among the MDD, PD, and HC groups measured during the stress and relaxation tasks.

Feature	Task	MDD (N = 41)	PD (*N* = 47)	HC (*N* = 59)	*F* (*P* value)	Post-hoc
RRI (s)	STR	0.777 ± 0.125	0.786 ± 0.124	0.784 ± 0.110	0.319 (.727)[a]	ns[a]
	RLX	0.831 ± 0.127	0.835 ± 0.134	0.857 ± 0.117	3.044 (.049)	None
SDNN (s)	STR	0.032 ± 0.014	0.032 ± 0.012	0.041 ± 0.016	32.543 (< .001)	MDD, PD < HC
	RLX	0.031 ± 0.015	0.031 ± 0.013	0.048 ± 0.062	10.446 (< .001)	MDD, PD < HC
RMSSD (s)	STR	0.021 ± 0.013	0.020 ± 0.009	0.028 ± 0.014	28.911 (< .001)	MDD, PD < HC
	RLX	0.021 ± 0.013	0.021 ± 0.011	0.034 ± 0.062	8.374 (< .001)[a]	MDD, PD < HC[a]
pNN50 (%)	STR	5.649 ± 9.643	3.598 ± 5.945	9.403 ± 11.742	24.865 (< .001)	PD < MDD < HC
	RLX	5.743 ± 10.493	4.064 ± 7.828	10.985 ± 12.630	27.117 (< .001)	MDD, PD < HC
TRI	STR	8.537 ± 3.323	8.679 ± 3.121	10.829 ± 3.703	33.248 (< .001)[a]	MDD, PD < HC[a]
	RLX	7.733 ± 3.088	7.938 ± 2.852	10.670 ± 3.925	50.638 (< .001)	MDD, PD < HC
TINN (s)	STR	0.145 ± 0.063	0.151 ± 0.058	0.187 ± 0.074	25.278 (< .001)	MDD, PD < HC
	RLX	0.135 ± 0.066	0.139 ± 0.059	0.191 ± 0.094	34.001 (< .001)	MDD, PD < HC
logVLF (s^2)	STR	-3.456 ± 6.494	-2.181 ± 7.014	-3.166 ± 6.493	1.837 (.161)	ns
	RLX	-3.482 ± 6.573	-1.987 ± 7.121	-2.847 ± 6.778	2.224 (.110)	ns
logLF (s^2)	STR	-3.652 ± 6.534	-2.377 ± 7.089	-3.299 ± 6.731	1.735 (.178)	ns
	RLX	-4.131 ± 6.636	-2.740 ± 7.191	-3.416 ± 6.769	1.883 (.153)	ns
LFnu (nu)	STR	65.184 ± 15.830	66.456 ± 14.107	64.417 ± 13.335	1.234 (.292)	ns
	RLX	55.220 ± 22.172	58.797 ± 18.939	56.453 ± 18.737	1.554 (.213)	ns
logHF (s^2)	STR	-4.361 ± 6.586	-3.133 ± 7.200	-3.948 ± 6.656	1.520 (.220)	ns
	RLX	-4.398 ± 6.618	-3.153 ± 7.209	-3.715 ± 6.765	1.521 (.220)	ns
HFnu (nu)	STR	34.692 ± 15.765	33.431 ± 14.039	35.460 ± 13.266	1.235 (.292)	ns
	RLX	44.685 ± 22.140	41.100 ± 18.920	43.442 ± 18.745	1.561 (.211)	ns
LF/HF	STR	2.708 ± 2.295	2.676 ± 1.953	2.326 ± 1.589	3.157 (.044)	None
	RLX	2.335 ± 3.041	2.211 ± 2.153	1.924 ± 1.743	2.046 (.131)	ns
logTot (s^2)	STR	-2.518 ± 6.517	-1.259 ± 7.040	-2.208 ± 6.589	1.751 (.175)	ns
	RLX	-2.669 ± 6.565	-1.302 ± 7.134	-2.027 ± 6.739	1.849 (.159)	ns
SD1 (s)	STR	0.015 ± 0.009	0.014 ± 0.007	0.020 ± 0.010	28.898 (< .001)	MDD, PD < HC
	RLX	0.015 ± 0.009	0.015 ± 0.008	0.024 ± 0.044	8.366 (< .001)[a]	MDD, PD < HC[a]
SD2 (s)	STR	0.042 ± 0.018	0.043 ± 0.017	0.055 ± 0.021	30.729 (< .001)	MDD, PD < HC
	RLX	0.040 ± 0.020	0.042 ± 0.017	0.062 ± 0.048	23.193 (< 0.001)	MDD, PD < HC
ApEn	STR	1.127 ± 0.118	1.124 ± 0.121	1.159 ± 0.090	8.382 (< 0.001)	MDD, PD < HC
	RLX	1.077 ± 0.105	1.092 ± 0.091	1.101 ± 0.108	3.115 (0.045)[a]	MDD < HC[a]
SampEn	STR	1.454 ± 0.297	1.428 ± 0.290	1.542 ± 0.275	10.417 (< 0.001)[a]	MDD, PD < HC[a]
	RLX	1.398 ± 0.275	1.403 ± 0.231	1.474 ± 0.241	7.213 (< 0.001)	MDD, PD < HC
α1	STR	1.225 ± 0.256	1.250 ± 0.220	1.202 ± 0.210	2.724 (0.067)	ns
	RLX	1.090 ± 0.321	1.134 ± 0.274	1.110 ± 0.239	1.014 (0.364)	ns
α2	STR	0.889 ± 0.219	0.853 ± 0.219	0.857 ± 0.206	1.696 (0.184)[a]	ns[a]
	RLX	0.979 ± 0.243	1.010 ± 0.194	0.952 ± 0.184	5.274 (0.006)	PD > HC
CorDim	STR	1.400 ± 1.529	1.296 ± 1.325	2.113 ± 1.496	22.515 (< 0.001)	MDD, PD < HC
	RLX	1.265 ± 1.424	1.035 ± 1.162	2.151 ± 1.446	45.805 (< 0.001)	MDD, PD < HC

Mean values computed from all the five visits were compared.

Data are presented as means and standard deviations. See **Supplementary Table 4** for post-hoc *P*-values.

STR: stress task; RLX: relaxation task; ns: no significant main effect; None: significant main effect but no significant differences in pair-wise comparisons; MDD: major depressive disorder; PD: panic disorder; HC: healthy control.

[a]Fisher's one-way ANOVA and Bonferroni post-hoc analysis were used. Except for these cases, Welch's ANOVA and Games-Howell post-hoc analysis were employed.

Supplementary Table 4. Post-hoc *P*-values for comparisons among the three groups shown in Supplementary Table 3.

Feature	Task	MDD vs. PD	MDD vs. HC	PD vs. HC
RRI (s)	RLX	.966	.076	.155
SDNN (s)	STR	.941	< .001	< .001
	RLX	.892	< .001	< .001
RMSSD (s)	STR	.400	< .001	< .001
	RLX[a]	1.00	.003	.001
pNN50 (%)	STR	.039	< .001	< .001
	RLX	.191	< .001	< .001
TRI	STR[a]	1.00	< .001	< .001
	RLX	.785	< .001	< .001
TINN (s)	STR	.688	< .001	< .001
	RLX	.788	< .001	< .001
LF/HF	STR	.989	.126	.101
SD1 (s)	STR	.400	< .001	< .001
	RLX[a]	1.00	.003	.001
SD2 (s)	STR	.797	< .001	< .001
	RLX	.780	< .001	< .001
ApEn	STR	.950	.006	.002
	RLX[a]	.487	.038	.964
SampEn	STR[a]	1.00	.004	< .001
	RLX	.984	.007	.004
α2	RLX	.374	.406	.004
CorDim	STR	.763	< .001	< .001
	RLX	.207	< .001	< .001

STR: stress task; RLX: relaxation task; MDD: major depressive disorder; PD: panic disorder; HC: healthy control.

[a]Fisher's one-way ANOVA and Bonferroni post-hoc analysis were used. Except for these cases, Welch's ANOVA and Games-Howell post-hoc analysis were employed.

Supplementary Table 5. Differences in HRV features between stress and relaxation tasks calculated within the same participant during a single visit (ΔHRV).

Feature	MDD		PD		HC	
	STR – RLX	*t* (*P* value)	STR – RLX	*t* (*P* value)	STR – RLX	*t* (*P* value)
ΔRRI (s)	-0.055 ± 0.045	-16.151 (< *.001*)	-0.049 ± 0.049	-13.681 (< *.001*)	-0.073 ± 0.055	-22.164 (< *.001*)
ΔSDNN (ms)[a]	1.099 ± 9.537	1.546 (.124)	0.873 ± 11.268	1.068 (.287)	-6.972 ± 61.149	-1.898 (.059)
ΔRMSSD (ms)[a]	-0.371 ± 5.779	-0.861 (.391)	-1.053 ± 6.329	-2.294 (*.023*)	-6.688 ± 60.998	-1.825 (.069)
ΔpNN50 (%)	-0.094 ± 5.396	-0.235 (.815)	-0.466 ± 4.652	-1.381 (.169)	-1.582 ± 8.520	-3.090 (*.002*)
ΔTRI	0.804 ± 2.314	4.662 (< *.001*)	0.741 ± 2.782	3.672 (< *.001*)	0.158 ± 3.381	0.780 (.436)
ΔTINN (ms)[a]	10.409 ± 45.138	3.094 (*.002*)	11.325 ± 52.199	2.990 (*.003*)	-4.442 ± 77.253	-0.957 (.339)
ΔlogVLF (s^2)	0.026 ± 1.462	0.240 (.811)	-0.194 ± 1.108	-2.412 (*.017*)	-0.319 ± 1.131	-4.694 (< *.001*)
ΔlogLF (s^2)	0.479 ± 1.356	4.741 (< *.001*)	0.364 ± 0.951	5.270 (< *.001*)	0.117 ± 0.900	2.163 (*.031*)
ΔLFnu (nu)	9.964 ± 19.325	6.918 (< *.001*)	7.658 ± 18.189	5.804 (< *.001*)	7.964 ± 19.387	6.837 (< *.001*)
ΔlogHF (s^2)	0.036 ± 1.226	0.397 (.692)	0.020 ± 0.642	0.421 (.674)	-0.233 ± 0.628	-6.170 (< *.001*)
ΔHFnu (nu)	-9.993 ± 19.306	-6.944 (< *.001*)	-7.669 ± 18.180	-5.815 (< *.001*)	-7.982 ± 19.369	-6.858 (< *.001*)
ΔLF/HF	0.372 ± 3.029	1.649 (.101)	0.465 ± 2.192	2.926 (*.004*)	0.401 ± 1.833	3.645 (< *.001*)
ΔlogTot (s^2)	0.151 ± 1.220	1.657 (.099)	0.044 ± 0.804	0.746 (.457)	-0.181 ± 0.821	-3.669 (< *.001*)
ΔSD1 (ms)[a]	-0.264 ± 4.092	-0.865 (.388)	-0.747 ± 4.483	-2.297 (*.023*)	-4.741 ± 43.231	-1.825 (.069)
ΔSD2 (ms)[a]	1.751 ± 13.773	1.706 (.090)	1.586 ± 15.994	1.367 (.173)	-6.868 ± 45.659	-2.504 (*.013*)
ΔApEn	0.050 ± 0.131	5.184 (< *.001*)	0.032 ± 0.124	3.528 (*.001*)	0.058 ± 0.131	7.346 (< *.001*)
ΔSampEn	0.056 ± 0.291	2.572 (*.011*)	0.025 ± 0.294	1.184 (.238)	0.068 ± 0.302	3.719 (< *.001*)
Δα1	0.134 ± 0.243	7.413 (< *.001*)	0.116 ± 0.222	7.173 (< *.001*)	0.092 ± 0.227	6.757 (< *.001*)
Δα2	-0.090 ± 0.276	-4.383 (< *.001*)	-0.157 ± 0.244	-8.895 (< *.001*)	-0.095 ± 0.250	-6.364 (< *.001*)
ΔCorDim	0.135 ± 1.073	1.691 (.093)	0.261 ± 1.053	3.411 (< *.001*)	-0.037 ± 1.181	-0.528 (.598)

A feature value measured during the relaxation task was subtracted from that during the stress task. Data are presented as the mean and standard deviation computed from all visits of all the participants. A paired t-test was used to compare two repeated measurements taken during the stress and relaxation tasks. *P*-values in italics < .05.

STR: stress task; RLX: relaxation task; MDD: major depressive disorder; PD: panic disorder; HC: healthy control.

[a]Five features, SDNN, RMSSD, TINN, SD1, and SD2, are presented in millisecond units.

Supplementary Table 6. Differences in the longitudinally scaled HRV features between the stress and relaxation tasks calculated within the same participant during a single visit (ΔHRV_{scaled}).

Feature (a.u.)	MDD		PD		HC	
	STR – RLX	*t*<br>(*P* value)	STR – RLX	*t*<br>(*P* value)	STR – RLX	*t*<br>(*P* value)
ΔRRI	-2.062 ± 0.949	-29.147<br>(*< 0.001*)	-1.668 ± 1.384	-16.610<br>(*< 0.001*)	-2.064 ± 0.926	-37.099<br>(*< 0.001*)
ΔSDNN	0.345 ± 1.518	3.046<br>(*0.003*)	0.181 ± 1.715	1.454<br>(0.148)	-0.319 ± 1.688	-3.144<br>(*0.002*)
ΔRMSSD	0.059 ± 1.845	0.428<br>(0.669)	-0.121 ± 1.753	-0.949<br>(0.344)	-0.776 ± 1.722	-7.504<br>(*< 0.001*)
ΔpNN50	0.125 ± 1.610	1.041<br>(0.299)	0.220 ± 1.577	1.926<br>(0.056)	-0.520 ± 1.686	-5.137<br>(*< 0.001*)
ΔTRI	0.575 ± 1.590	4.855<br>(*< 0.001*)	0.524 ± 1.758	4.109<br>(*< 0.001*)	0.175 ± 1.759	1.652<br>(0.100)
ΔTINN	0.458 ± 1.595	3.856<br>(*< 0.001*)	0.385 ± 1.691	3.139<br>(*0.002*)	-0.025 ± 1.711	-0.240<br>(0.811)
ΔlogVLF	-0.133 ± 1.536	-1.166<br>(0.245)	-0.358 ± 1.742	-2.832<br>(*0.005*)	-0.489 ± 1.648	-4.933<br>(*< 0.001*)
ΔlogLF	0.695 ± 1.547	6.030<br>(*< 0.001*)	0.625 ± 1.585	5.436<br>(*< 0.001*)	0.131 ± 1.668	1.310<br>(0.191)
ΔLFnu	0.694 ± 1.638	5.682<br>(*< 0.001*)	0.647 ± 1.602	5.572<br>(*< 0.001*)	0.533 ± 1.671	5.311<br>(*< 0.001*)
ΔlogHF	-0.098 ± 1.834	-0.716<br>(0.475)	-0.169 ± 1.788	-1.305<br>(0.193)	-0.728 ± 1.636	-7.406<br>(*< 0.001*)
ΔHFnu	-0.695 ± 1.637	-5.699<br>(*< 0.001*)	-0.650 ± 1.603	-5.588<br>(*< 0.001*)	-0.535 ± 1.671	-5.331<br>(*< 0.001*)
ΔLF/HF	0.719 ± 1.635	5.903<br>(*< 0.001*)	0.673 ± 1.607	5.775<br>(*< 0.001*)	0.609 ± 1.673	6.055<br>(*< 0.001*)
ΔlogTot	0.165 ± 1.571	1.407<br>(0.161)	0.040 ± 1.693	0.325<br>(0.745)	-0.344 ± 1.664	-3.442<br>(*< 0.001*)
ΔSD1	0.058 ± 1.845	0.425<br>(0.672)	-0.121 ± 1.753	-0.953<br>(0.342)	-0.777 ± 1.722	-7.512<br>(*< 0.001*)
ΔSD2	0.349 ± 1.512	3.097<br>(*0.002*)	0.191 ± 1.714	1.533<br>(0.127)	-0.285 ± 1.672	-2.834<br>(*0.005*)
ΔApEn	0.823 ± 1.579	6.991<br>(*< 0.001*)	0.648 ± 1.655	5.398<br>(*< 0.001*)	0.949 ± 1.522	10.369<br>(*< 0.001*)
ΔSampEn	0.352 ± 1.673	2.825<br>(*0.005*)	0.196 ± 1.669	1.619<br>(0.107)	0.411 ± 1.721	3.978<br>(*< 0.001*)
Δα1	0.840 ± 1.588	7.095<br>(*< 0.001*)	0.781 ± 1.518	7.097<br>(*< 0.001*)	0.621 ± 1.594	6.479<br>(*< 0.001*)
Δα2	-0.573 ± 1.663	-4.622<br>(*< 0.001*)	-0.895 ± 1.462	-8.436<br>(*< 0.001*)	-0.608 ± 1.588	-6.375<br>(*< 0.001*)
ΔCorDim	0.275 ± 1.676	2.204<br>(*0.029*)	0.441 ± 1.795	3.384<br>(*< 0.001*)	0.040 ± 1.875	0.351<br>(0.726)

A feature value measured during the relaxation task is subtracted from that during the stress task. Data are presented as the mean and standard deviation computed from all visits of all the participants. A paired t-test was used to compare two repeated measurements taken during stress and relaxation tasks. *P*-values in italics < .05.

STR: stress task; RLX: relaxation task; MDD: major depressive disorder; PD: panic disorder; HC: healthy control.

Supplementary Table 7. Performance measures for classifying the stress and relaxation tasks via a MLP classifier.

Scaling	**Pre-longitudinal scaling**				**Post-longitudinal scaling**			
Group	Overall	MDD	PD	HC	Overall	MDD	PD	HC
Accuracy	0.6875 ± 0.0079	0.6630 ± 0.0154	0.6810 ± 0.0169	0.7085 ± 0.0114	0.9318 ± 0.0050	0.9392 ± 0.0060	0.8971 ± 0.0110	0.9501 ± 0.0056
F1	0.6761 ± 0.0100	0.6557 ± 0.0191	0.6581 ± 0.0193	0.7074 ± 0.0107	0.9315 ± 0.0050	0.9383 ± 0.0062	0.8961 ± 0.0113	0.9506 ± 0.0055
Recall	0.6622 ± 0.0168	0.6428 ± 0.0353	0.6144 ± 0.0284	0.7049 ± 0.0137	0.9287 ± 0.0066	0.9238 ± 0.0101	0.8874 ± 0.0141	0.9604 ± 0.0073
Precision	0.7010 ± 0.0111	0.6706 ± 0.0196	0.7097 ± 0.0240	0.7102 ± 0.0140	0.9356 ± 0.0068	0.9533 ± 0.0088	0.9050 ± 0.0109	0.9410 ± 0.0077
AUC	0.7581 ± 0.0107	0.7260 ± 0.0137	0.7387 ± 0.0173	0.7693 ± 0.0169	0.9741 ± 0.0037	0.9773 ± 0.0057	0.9450 ± 0.0078	0.9835 ± 0.0032

Performance measures are calculated separately for each patient group in the test dataset in addition to the overall evaluation based on the entire test data.

Results are presented as mean and standard deviation calculated from the 20 repeats.

AUC: area under the curve; MDD: major depressive disorder; PD: panic disorder; HC: healthy control.

2 Supplementary Figures

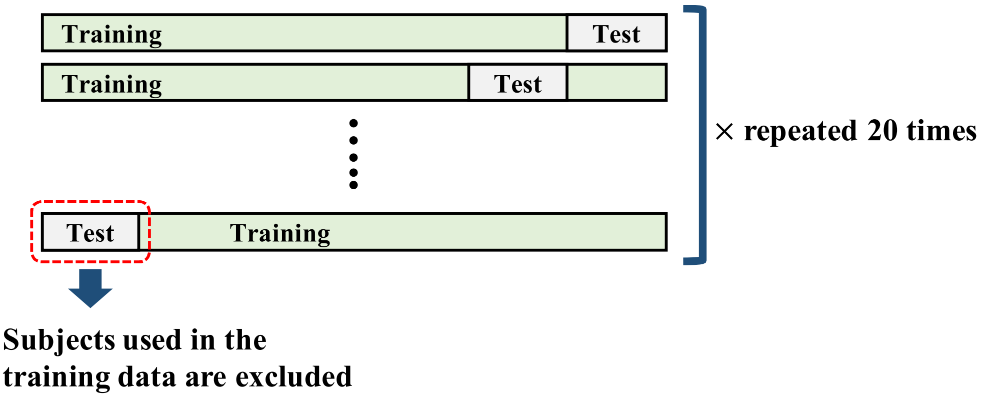

Supplementary Figure 1. Overview of the repeated K-fold cross-validation method. We used a 10-fold cross-validation approach repeated 20 times. During each fold, the participants included in the training data were excluded from the test data to prevent data leakage.

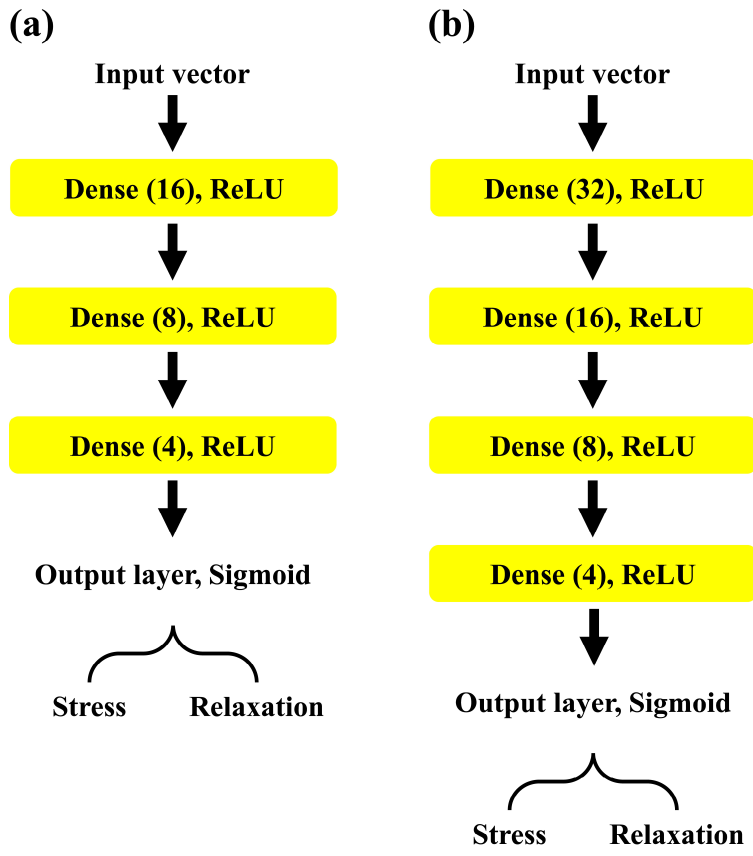

Supplementary Figure 2. Architecture of the (a) three-hidden-layer and (b) four-hidden-layer models.

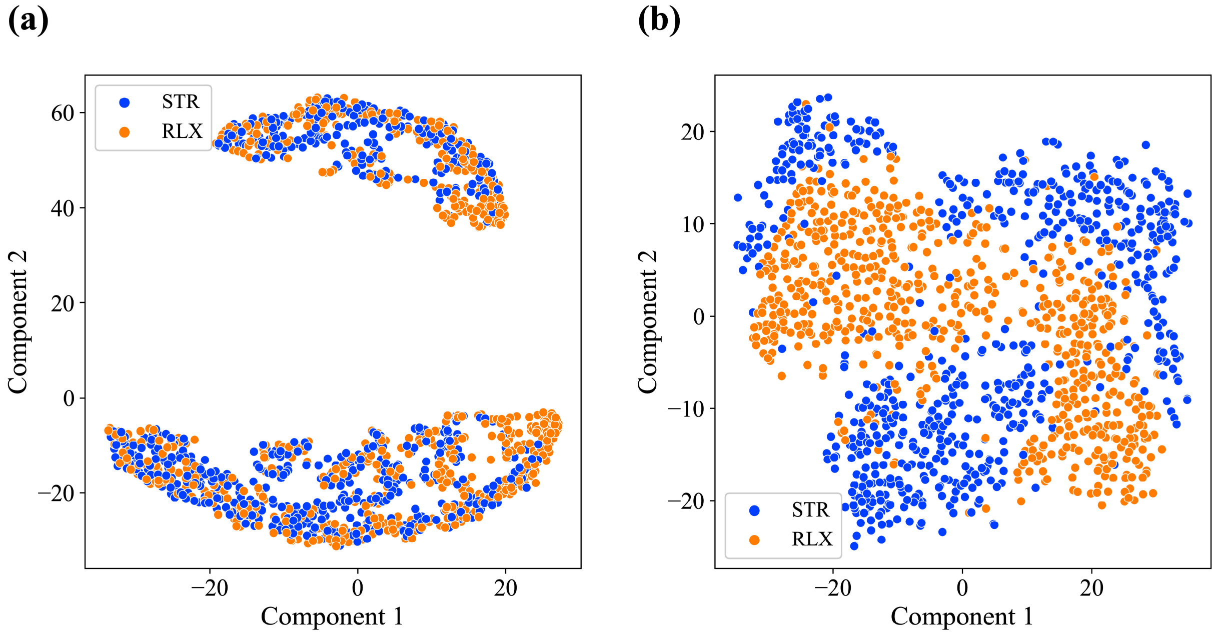

Supplementary Figure 3. Visualization of the stress and relaxation classes via t-SNE based on (a) original HRV data and (b) longitudinally scaled HRV data.
